# Nintedanib Inhibits Wnt3a-Induced Myofibroblast Activation by Suppressing the Src/β-Catenin Pathway

**DOI:** 10.3389/fphar.2020.00310

**Published:** 2020-03-16

**Authors:** Xiaohe Li, Xiaowei Liu, Ruxia Deng, Shaoyan Gao, Haiyan Yu, Kai Huang, Qiuyan Jiang, Rui Liu, Xiaoping Li, Liang Zhang, Honggang Zhou, Cheng Yang

**Affiliations:** ^1^ State Key Laboratory of Medicinal Chemical Biology, College of Pharmacy and Tianjin Key Laboratory of Molecular Drug Research, Nankai University, Tianjin, China; ^2^ Tianjin Key Laboratory of Molecular Drug Research, Tianjin International Joint Academy of Biomedicine, Tianjin, China; ^3^ Department of Thoracic Surgery, Tian Jin First Central Hospital, Tianjin, China

**Keywords:** Nintedanib, Wnt/β-catenin, Src kinase, pulmonary fibrosis, myofibroblast

## Abstract

Idiopathic pulmonary fibrosis (IPF) is an interstitial lung disease characterized by epithelial cell damage, myofibroblast activation, and collagen deposition. Multiple studies have documented that the Wnt/β-catenin pathway is aberrantly activated in IPF and plays a vital role in myofibroblast differentiation and activation. Kinases such as Src initiate Wnt/β-catenin signaling by phosphorylating β-catenin at tyrosine residues, which facilitates β-catenin accumulation in the nucleus and promotion of fibrosis progression. Nintedanib has been approved for the treatment of IPF as a multitargeted tyrosine kinase inhibitor. Nintedanib has been demonstrated to directly block Src, and whether it attenuates pulmonary fibrosis through regulating the Wnt/β-catenin pathway remains unclear. In this study, we found that nintedanib attenuated myofibroblast activation through inhibiting the expression of genes downstream of Wnt signaling such as Cyclin D1, Wisp1, and S100a4. Further experiments showed that nintedanib inhibited Wnt3a-induced β-catenin nuclear translocation through suppressing Src kinase activation and β-catenin Y654 phosphorylation. Additionally, Src knockdown fibroblasts exhibited a phenotype similar to that of the nintedanib treatment group, and the inhibitory effects of nintedanib were consistent with those of the Src kinase inhibitor KX2-391. In summary, our study shows that nintedanib exhibits an anti-fibrosis effect, partly by inhibiting the Src/β-catenin pathway.

## Introduction

Idiopathic pulmonary fibrosis (IPF) is a progressive, chronic fibrosing interstitial pneumonia with an unknown etiology that occurs primarily in older adults (Lynch et al., 2000). The annual incidence of IPF in the USA population was estimated to be 6.8–8.8 per 100,000 using narrow case definitions, while in Europe, it was 0.22–7.4 per 100,000 (Nalysnyk et al., 2012). IPF has a poor prognosis, with a median survival time of 3.8 years in the USA (Raghu et al., 2014). Recent research has indicated that IPF has a multivariate etiology that incorporates genetic susceptibility, epigenetics, ageing, and the environment (Thannickal et al., 2014). IPF is characterized by lung architecture destruction, aberrant activation of myofibroblasts and extracellular matrix (ECM) deposition (Martinez et al., 2017). Myofibroblasts are primary effector cells in pulmonary fibrosis progression and exhibit a higher proliferation rate and contractile phenotype, causing overexpression of fibronectin (Fn), collagen I (Col I), and α-smooth muscle actin (α-SMA) (Sgalla et al., 2018).

Several signaling pathways are thought to play specific roles in fibrogenesis.

Wnt/β-catenin signaling has been shown to be a major regulatory pathway in adult organ reparation (Chilosi et al., 2003; Konigshoff et al., 2008). In the canonical Wnt/β-catenin pathway, ligand binding induces the accumulation of cytoplasmic β-catenin in the nucleus, resulting in the binding of β-catenin to transcriptional binding sequence T-cell factor (TCF)/lymphocyte enhance factor (LEF) and the regulation of downstream gene transcription (Grainger and Willert, 2018). Under homeostasis, β-catenin is recruited to the membrane by interacting with E-cadherin (E-Cad) to respond to cell-cell or cell-matrix contact signals (Richeldi et al., 2014). Once stimulated by hepatocyte growth factor (HGF) (Gayrard et al., 2018) or transforming growth factor-β (TGF-β) (Kim et al., 2009), the β-catenin Y654 residue is phosphorylated by Src kinase; then, it dissociates from E-Cad and translocates into the nucleus where it promotes fibrogenesis (Kim et al., 2009; Ulsamer et al., 2012).

Nintedanib is an effective small molecule tyrosine kinase inhibitor that inhibits proteins in three major signaling angiogenesis pathways, including the vascular endothelial growth factor receptor (VEGFR) family, the fibroblast growth factor receptor (FGFR) family, and the platelet-derived growth factor receptor (PDGFR) family (Hilberg et al., 2008; Roth et al., 2015). It was approved as an anti-IPF drug by the FDA in 2014 and is recommended by international guidelines and consensus to treat pulmonary fibrosis. Clinical studies have shown that nintedanib improves the forced vital capacity (FVC) of patients (Richeldi et al., 2014; Kolb et al., 2017). *In vitro* studies have also revealed that it has signiﬁcant effects on the suppression of myofibroblast proliferation, migration, and transformation through blocking tyrosine kinases (Dimitroulis, 2014; Hostettler et al., 2014). In addition to its strong effects as a tyrosine kinases inhibitor, nintedanib has also been found to exert several inhibitory effects, including effects on JNK/AP-1 (Kamio et al., 2015), TGF-β (Rangarajan et al., 2016), and the PHMG-induced inflammatory response (Kim et al., 2018). Nintedanib is an excellent Src kinase inhibitor (Hilberg et al., 2008); however, there is no evidence to support whether such Src kinase blockage would affect factors downstream of the Wnt/β-catenin pathway. In this study, we demonstrate that nintedanib can inhibit Wnt3a-induced myofibroblast activation through inhibiting Src kinase activity.

## Materials and Methods

### Reagents

Nintedanib (>99%) was purchased from HWRK Chem Co., Ltd. (Beijing, China). For each experiment, nintedanib was freshly prepared by dissolving it in DMSO (Sigma-Aldrich, USA). Bleomycin (BLM) was acquired from Nippon Kayaku (Tokyo, Japan). Wnt3a was purchased from Peprotech (Texas, USA). TRIzol reagent and DEPC-treated water were obtained from Thermo Fisher Scientific corporation (Waltham, USA). RNase, DNase, and DNA Away H_2_O were purchased from Beyotime Biotechnology (Beijing, China). FastKing gDNA Dispelling RT SuperMix was obtained from Tiangen Biotech Co., Ltd. (Beijing, China). The inhibitor KX2-391, RIPA lysis buffer (middle) and the BCA kit were purchased from Beyotime Biotechnology (Beijing, China). The primary antibodies described in the study included anti-fibronectin, anti-collagen I, and anti-β-tubulin antibodies (Affinity Biosciences, USA); anti-GAPDH, anti-p(Y416)-Src, and anti-Src antibodies (Cell Signalling Technology, USA); anti-α-actin antibody (Santa Cruz Biotechnology, USA); anti-β-catenin and anti-lamin B1 antibodies (ProteinTech Group, China); and anti-p(Y654)-β-catenin antibody (Immunoway Biotechnology, China). The secondary antibodies HRP-labeled goat anti-rabbit IgG and HRP-labeled goat anti-mouse IgG were from Abcam (Cambridge, UK).

### Cell Culture

The mouse lung myofibroblast cells line (Mlg) were grown in DMEM (Solarbio, China) supplemented with 10% FBS (ExCellBio, China). Cells were maintained at 37°C with 5% CO_2_ in a humidified atmosphere.

### Isolation of Primary Pulmonary Myofibroblasts

Primary pulmonary myofibroblasts (PPF) isolated from male C57BL/6 J mice were cultured in DMEM supplemented with 10% FBS at 37°C with 5% CO_2_ in a humidified atmosphere as described previously (Jiang et al., 2005). Cells at passages 3-4 were used for RT-PCR and western blotting assays.

### Animals and BLM Administration

Six- to eight-week-old male C57BL/6 J mice were obtained from the Laboratory Animal Centre, Academy of Military Medical Sciences of People's Liberation Army (Beijing, China). A total of 18 mice weighing between 20–23 g were used in the experiments. The mice were housed under controlled temperature (22–26°C) and a 12-h light-dark cycle. All animal care and experimental procedures complied with guidelines approved by the Institutional Animal Care and Use Committee (IACUC) of Nankai University (Permit No. SYXK 2014-0003). Animal studies are reported in compliance with the ARRIVE guidelines (Kliment and Oury, 2010; McGrath and Lilley, 2015).

Intratracheal BLM administration was performed as described previously (Jiang et al., 2004). In brief, the mice were anaesthetized with an intraperitoneal injection of 10% chloral hydrate and then intratracheally injected with 2 mg/kg BLM (Blenoxane, Nippon Kayaku Co., Ltd) using a sterile insulin syringe. After injection, the animals were immediately erected and rotated left and right to distribute the drug solution evenly in the lungs. In the sham operation group, the same amount of saline was injected intratracheally using the same method. Eighteen mice were randomly divided into three groups with six animals per group: control group, BLM group, and BLM+nintedanib group (100 mg·kg^−1^). Nintedanib was intragastrically administered daily for 1 week beginning 7 days after BLM injury. The control and model groups received an equal volume of vehicle (0.5% CMC-Na) using the same schedule and route of administration. Mice were sacrificed at day 14 after BLM administration for the evaluation of pulmonary fibrosis.

### Nintedanib Treatment *In Vitro*

For *in vitro* experiments, nintedanib was used at two concentrations (1 and 2 μM) according to published studies and MTT assays (Hostettler et al., 2014; Huang et al., 2016).

### Dual Luciferase Assay

TCF/LEF promoters were cloned into the pGL4.49 luciferase reporter vector (Promega, USA), and Mlg cells were transfected with luciferase reporter plasmids using PEI (Polysciences, USA) to form the TCF/LEF-firefly luciferase (TOPFlash) assay system. Renilla-luciferase was used as an internal control. Cells were treated 18 h after transfection with a series of nintedanib for 8 h. The luciferase activity of cell lysates was determined using a luciferase assay system (Promega, USA) as described by the manufacturer. Total light emission was measured using a GloMax^®^-Multi Detection System (Promega, USA). All luciferase results were obtained from three independent experiments in different times, and the ordinate of the scatter plot represents the ratio of firefly-luciferase to Renilla-luciferase.

### Quantitative Real-Time Polymerase Chain Reaction (qRT-PCR)

We performed RNA isolation as previously described (Geng et al., 2011). Total RNA was extracted from cells and tissue using TRIzol Reagent. The cDNA was obtained by reverse transcription from total RNA using FastKing gDNA Dispelling RT SuperMix (TIANGEN, China). qRT-PCR was performed using Hieff UNICON Power qPCR SYBR Green Master Mix (Yeasen Biotech, China) according to the manufacturer's protocol. The specificity of this assay was confirmed using melting-curve analysis (**Figure S1**). β-Actin was used as the endogenous reference gene in all RT-PCR experiments. The quantification of gene expression was performed relative to an endogenous reference gene (*β-actin*) using the −ΔΔCT (cell samples) or −ΔCT (tissue samples) method in the experiments (Schmittgen and Livak, 2008).

(1) ΔCT=CT gene of interest−CT endogenous reference gene

(2) ΔΔCT=ΔCT treatment group−ΔCT control (CTL)group

Sequences of the specific primer sets are presented in **Table 1**. All cell simples were obtained from three independent experiments and qRT-PCR experiments were run on different plate at different times.

**Table 1:** Mouse primer sequences. Sequences were obtained from GenBank, and all accession numbers are indicated.

Gene	Accession		Sequences (5´ → 3´)	Length
α-SMA	NM_007392	for	GCTGGTGATGATGCTCCCA	19 bp
		rev	GCCCATTCCAACCATTACTCC	21 bp
Co1 Ia1	NM_007742	for	CCAAGAAGACATCCCTGAAGTCA	23 bp
		rev	TGCACGTCATCGCACACA	18 bp
Fn	NM_010233	for	GTGTAGCACAACTTCCAATTACGAA	25 bp
		rev	GGAATTTCCGCCTCGAGTCT	20 bp
β-actin	NM_007393	for	AGGCCAACCGTGAAAAGATG	20 bp
		rev	AGAGCATAGCCCTCGTAGATGG	22 bp
CCND1	NM_007631	for	GCGTACCCTGACACCAATCT	20 bp
		rev	CAGGTCTCCTCCGTCTTGAG	20 bp
Wisp1	NM_018865	for	CAGCACCACTAGAGGAAACGA	21 bp
		rev	CTGGGCACATATCTTACAGCATT	23 bp
S100a4	NM_011311	for	TGAGCAACTTGGACAGCAACA	21 bp
		rev	CTTCTTCCGGGGCTCCTTATC	21 bp

### Western Blot

The proteins were extracted from cells or lung tissues following standard protocols as described previously (Ning et al., 2004). Cells were lysed in radio-immunoprecipitation assay (RIPA) lysis buffer containing proteinase inhibitors to obtain total protein or with the Nuclear and Cytoplasmic Protein Extraction Kit (Beyotime, China) and then assayed with the BCA kit to determine the protein concentration. After electrophoresis and membrane transfer, the immunoblots were probed with the following specific primary antibodies: α-SMA, collagen I, fibronectin, p(Y416)-Src, Src, β-tubulin, p(Y654)-β-catenin, β-catenin, GAPDH, β-tubulin, and lamin B1. The secondary antibodies were goat anti-rabbit or goat anti-mouse horseradish peroxidase-conjugated antibody. The ECL Kit (Affinity Biosciences, USA) was used for detection, and the blots were scanned using ImageJ 8.0 software (**Data Sheets 1** and **2**). In the analysis of cell samples, GAPDH and lamin B1 were used as the endogenous reference protein and the relative expression values in treatment samples were normalized to CTL sample in each independent experiment. In the analysis of lung tissue samples, β-tubulin was used as the endogenous reference protein and relative expression of target proteins in each mouse was reflected by the ratio of phosphorylated protein to total protein. All western bot results were obtained from three independent experiments in different times.

### Immunofluorescence Staining

Cells were cultured in a 48-well chamber prior to immunofluorescent staining. After treatment with nintedanib, the cells were fixed in 4% paraformaldehyde for 20 min at room temperature. After washing three times with PBS, the cells were permeabilized with 0.2% Triton X-100 (Sigma-Aldrich, USA) for 10 min and blocked with 5% BSA for 60 min in a humidified chamber. Mlg cells were incubated with α-SMA antibody (1:100 dilutions) and β-catenin (1:200 dilutions) overnight at 4°C, respectively. After washing three times with PBS, the cells were incubated with fluorescein (FITC) AffiniPure goat anti-mouse IgG (H+L) or rhodamine (TRITC) AffiniPure goat anti-rabbit IgG (H+L) (Jackson ImmunoResearch, USA). Cell nuclei were labeled with DAPI (Solarbio, China), and the cells were photographed with a TCS SP8 confocal (Leica, Germany) microscope.

### siRNA Transfection

The siRNA duplexes targeting mouse Src mRNA were obtained from Tsingke Biology Technology (Beijing, China). The siRNA sequence was 5'-GAAGCUGAGGCAUGAGAAG-3'.

The siRNAs (final concentration 1 pmol) were transiently transfected into Mlg cells using Lipofectamine^®^ RNAiMAX Reagent (Invitrogen, USA) as described by the manufacturer. Cells were harvested and analyzed for mRNA and luciferase activity 48 h after transfection.

### Data and Statistical Analysis

The data and statistical analysis complied with the recommendations for the experimental design and analysis in pharmacology (Curtis et al., 2015) (**Data Sheet 3**). The data were processed with Prism 6.0 software and expressed as the mean ± SD. The significance differences between CTL and Wnt3a groups in Western blot experiments of cell samples were evaluated by one-sided one sample t-test, and the rest experiments were evaluated by pairwise t-test with subsequent Bonferroni correction. Results were considered statistically significant at P < 0.05.

### Nomenclature of Targets and Ligands

Key protein targets and ligands in this article are hyperlinked to corresponding entries at http://www.guidetopharmacology.org, the common portal for data from the IUPHAR/BPS Guide to PHARMACOLOGY (Southan et al., 2016), and they are permanently archived in the Concise Guide to PHARMACOLOGY 2015/16 (Alexander et al., 2015).

## Results

### Nintedanib Suppressed Wnt3a-Induced Myofibroblast Activation

**Figure 1:**
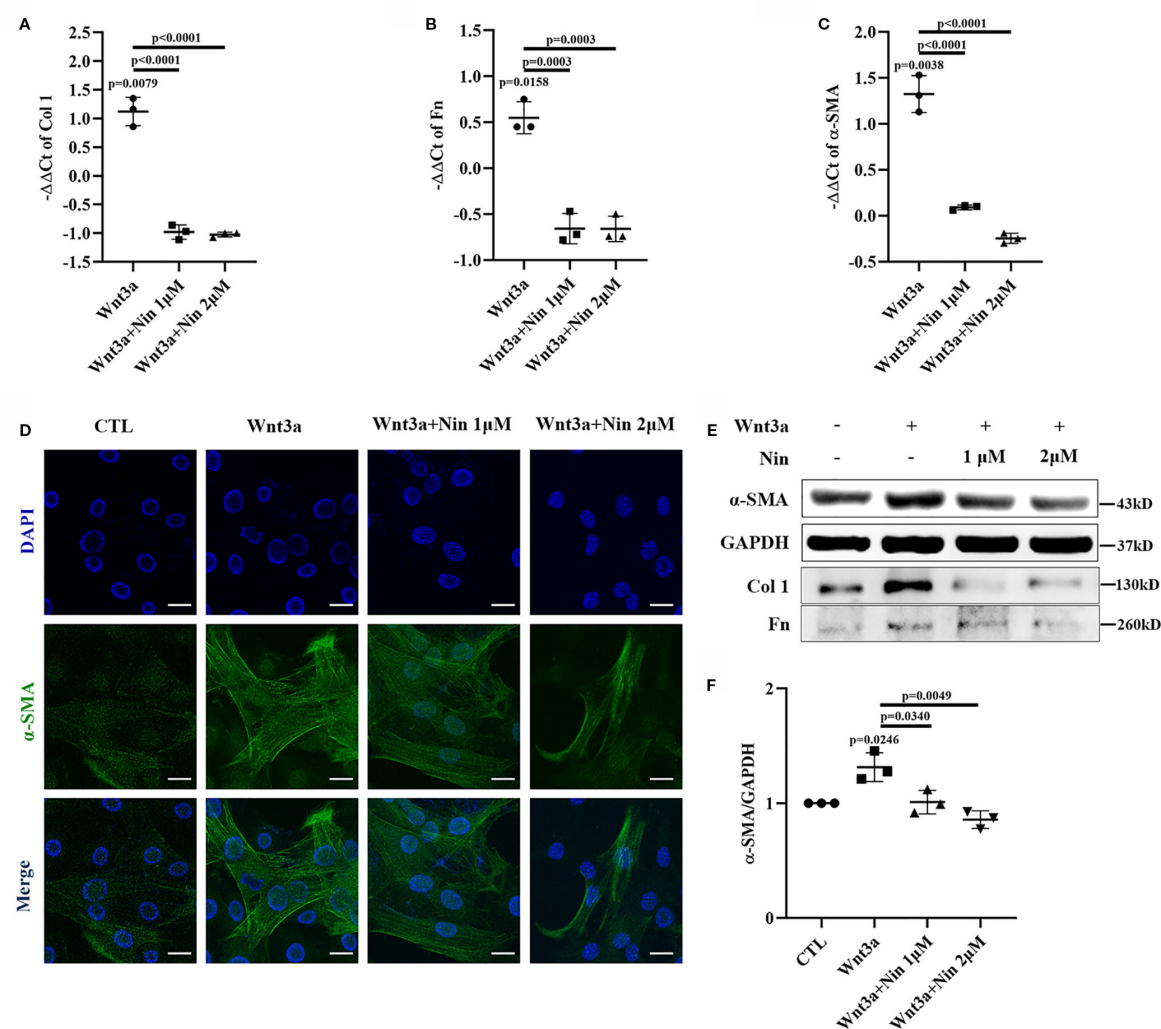
Nintedanib suppressed Wnt3a-mediated myofibroblast activation. Mlg cells were treated with Wnt3a (100 ng/ml) in the presence or absence of nintedanib (1 and 2 μM) for 24  h. **(A–C)** The mRNA levels of collagen I **(A)**, fibronectin **(B)**, and α-SMA **(C)** were examined by real-time PCR and β-actin was used as the endogenous reference gene. **(D)** The expression of α-SMA was detected by immunofluorescence. Nuclei were stained with DAPI (blue). Scale bar, 40 µm. **(E)** The protein levels of collagen I, fibronectin, and α-SMA were analysed by western blotting. Representative gel electrophoresis bands are shown, and **(F)** the expression levels of α-SMA were quantified by densitometry and GAPDH was used as the endogenous reference protein. Each bar represents the mean ± SD of triplicate independent experiments.

To explore the potential anti-fibrotic mechanism of nintedanib, we established a Wnt3a-induced myofibroblast activation model with mouse lung myofibroblast cells (Mlg) and treated Mlg with different doses of nintedanib. Next, we determined the RNA levels of the following activation makers: Col I, Fn, and α-SMA. The results showed that the expression of Col I, Fn, and α-SMA induced by Wnt3a was significantly decreased by nintedanib (**Figures 1A–C**).

We also performed immunofluorescence assays for α-SMA and observed fewer α-SMA positive-staining myofibroblasts among the nintedanib-treated cells (**Figure 1D**). Furthermore, western blotting assays indicated that nintedanib decreased the protein levels of Col I, Fn, and α-SMA (**Figures 1E, F**).

### Nintedanib Inhibited the Expression of Genes Downstream of the Wnt Signaling Pathway

**Figure 2:**
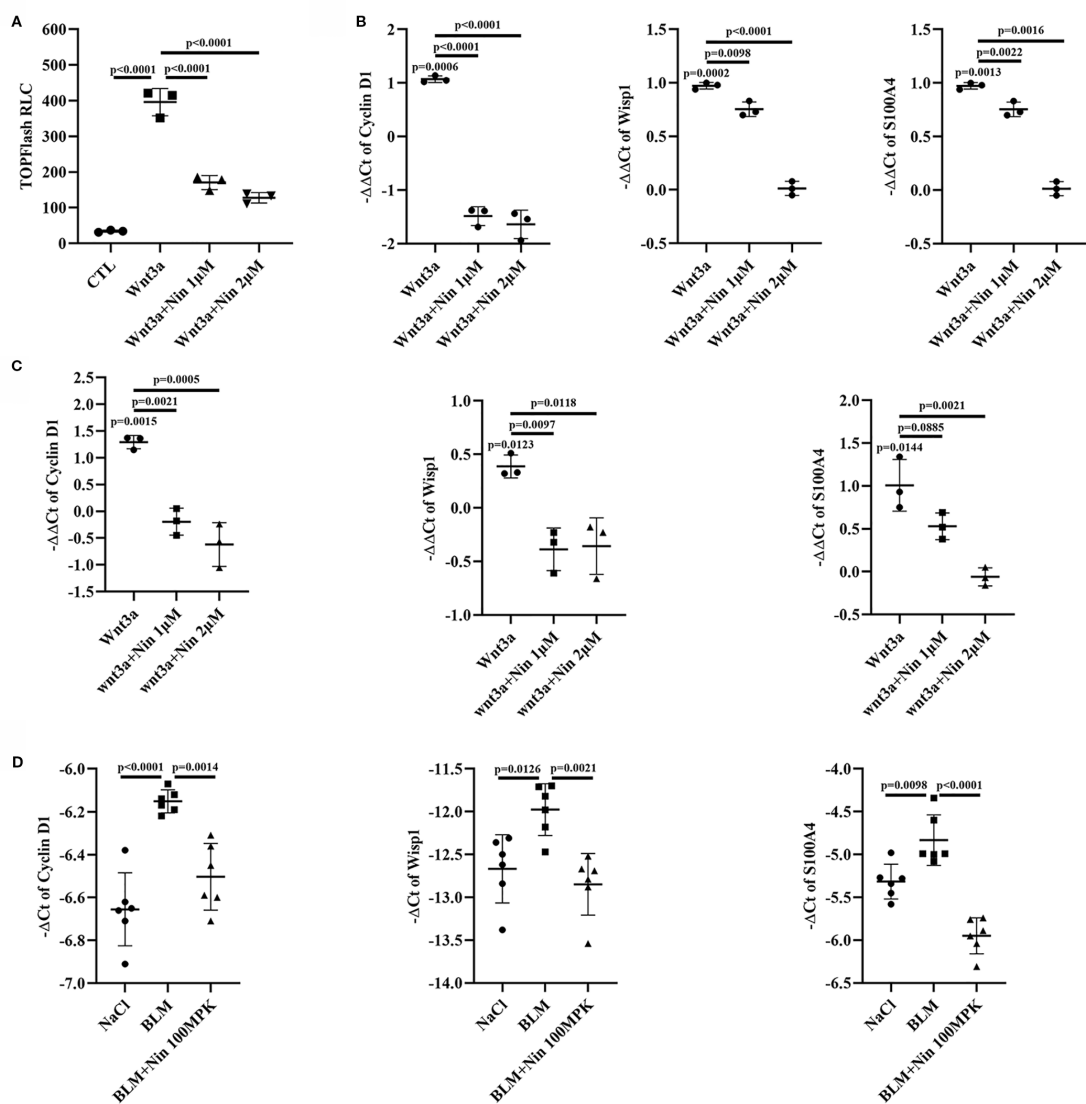
Nintedanib inhibited Wnt/β-catenin signaling. **(A)** The Mlg cells were transfected with the TOPFlash plasmids. After 18 h of transfection, the cells were treated with Wnt3a and nintedanib (1 and 2 μM) for 8 h and then lysed for luciferase assays. RLC, relative luciferase count. **(B–D)** The expression of genes downstream of Wnt signaling (Cyclin D1, Wisp1, and *S100a4*) was determined in Mlg (n = 3) **(B)**, PPF (n = 3) **(C)** and lung tissues (n = 6) **(D)** by quantitative real-time PCR. β-actin was used as the endogenous reference gene. Each bar represents the mean ± SD of triplicate independent experiments.

To further confirm the inhibitory effect of nintedanib on the Wnt/β-catenin pathway, we used the TOPFlash assay system and found that nintedanib inhibited Wnt3a-induced transcriptional activity in Mlg cells (**Figure 2A**). The endogenous Wnt target genes CyclinD1, Wisp1, and S100a4 were inhibited by nintedanib in Mlg and PPF cells, as determined by real-time PCR (**Figures 2B, C**). The attenuation of Wnt pathway activation by nintedanib was further supported by decreased mRNA levels of cyclinD1, Wisp1, and S100a4 *in vivo* (**Figure 2D**).

**Figure 3:**
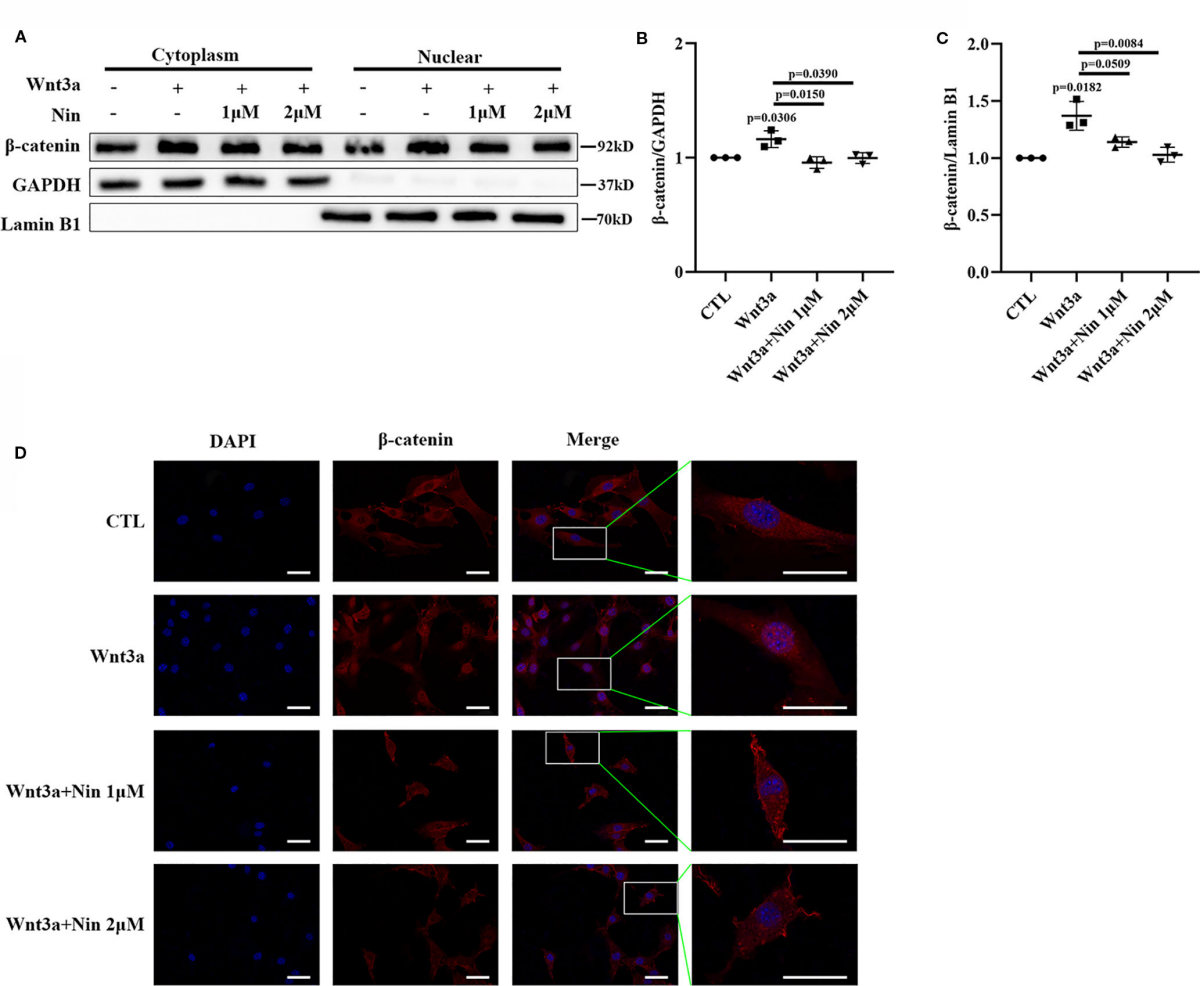
Nintedanib inhibited Wnt3a-induced nuclear translocation of β-catenin. Mlg cells were incubated with nintedanib (1 and 2 μM) for 2 h, and then, the cells were treated with Wnt3a for an additional 2 h. **(A)** The cells were fractionated as described in the Materials and Methods section, and the samples were analysed by western blotting. GAPDH was used as a cytosolic marker, and lamin B1 was used as a nuclear marker. **(B, C)** The immunoblots were quantified by densitometry. **(D)** β-catenin localization was detected by immunofluorescence. Scale bar, 60 µm. Each bar represents the mean ± SD of triplicate independent experiments.

As described above, the accumulation of nuclear β-catenin is a hallmark for the activation of canonical Wnt signaling (Mikels and Nusse, 2006). The potency of nintedanib inhibition in Wnt-mediated β-catenin stabilization was determined with immunoblots. **Figures 3A–C** shows that nuclear β-catenin levels were decreased in Mlg cells. This reduction in protein levels was further confirmed by immunofluorescence (**Figure 3D**). Collectively, the above data demonstrated that nintedanib inhibited myofibroblast activation by repressing Wnt/β-catenin pathway activity.

### Nintedanib Suppressed Wnt-Induced Src Activation and β-Catenin Phosphorylation

Src kinase has previously been shown to be required for β-catenin phosphorylation at tyrosine residue 654 (Y654) and the initiation of EMT, and it has been correlated with increased β-catenin-dependent transcription in both A549 cells and primary AECs (Kim et al., 2009; Ulsamer et al., 2012). As mentioned previously, nintedanib signiﬁcant inhibits Src family kinases (Li et al., 2017). Thus, we suspected that nintedanib could affect Wnt signaling through inhibiting Src activation in myofibroblasts.

**Figure 4:**
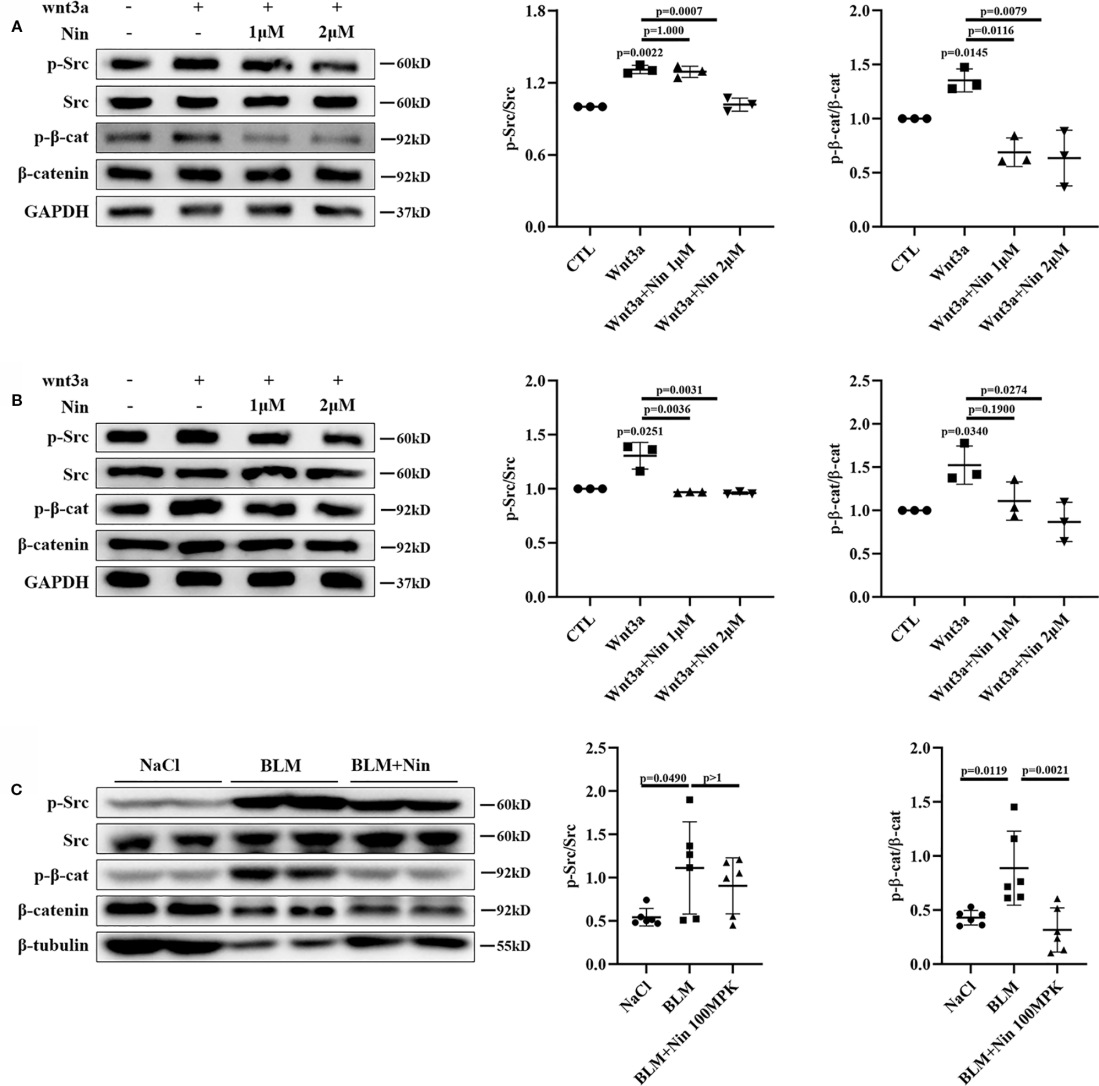
Nintedanib suppressed Wnt-induced Src activation and Y654-β-catenin phosphorylation. **(A, B)** Mlg **(A)** and PPF **(B)** cells were maintained in the presence of nintedanib (1 and 2 μM) for 2 h and then treated with Wnt3a for 2 h. Next, pY416-Src, total Src, pY654-β-catenin, and total β-catenin were evaluated by western blot analysis. Relative density of the bands of pY416-Src and pY654-β-catenin compared to Src or β-catenin is represented (n = 3). **(C)** Western blot analysis of pY654-β-catenin, total β-catenin, pY416-Src, total Src, and GAPDH in lung tissues of the different groups. Immunoblots of pY416-Src and pY654-β-catenin were quantified by densitometry (n = 6). Each bar represents the mean ± SD of triplicate independent experiments.

To verify this hypothesis, we detected the protein levels of pY416-Src to investigate Src activation. Simultaneously, pY654-β-catenin was tested in Wnt3a-treated Mlg and PPF cells. As expected, nintedanib reduced Wnt-induced Src activation and β-catenin phosphorylation *in vitro* (**Figures 4A, B**). Moreover, *in vivo* experiments showed the administration of nintedanib attenuated BLM-induced pY654-β-catenin (**Figure 4C**).

### Nintedanib Inhibited Wnt/β-Catenin Signaling Partly Through Deactivating Src Kinase

**Figure 5:**
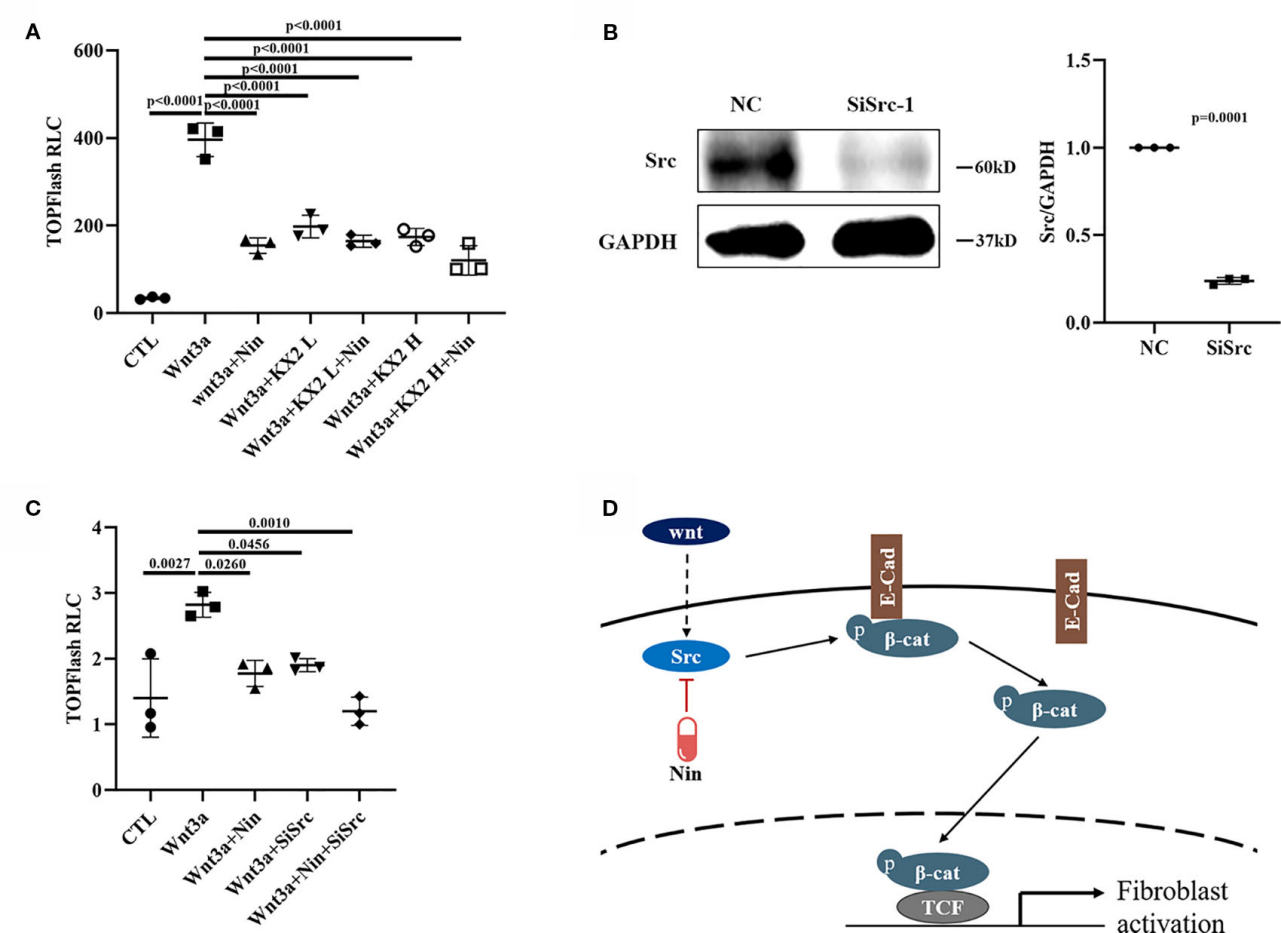
Nintedanib affected Wnt/β-catenin signaling partly through Src. **(A)** The Mlg cells were transfected with the TOPFlash plasmids for 18 h followed by treatment with Wnt3a (100 ng/ml), nintedanib (2 μM), and KX2-391 (1 and 2 μM) for 8 h and then lysis for luciferase assays. The KX2 L means low concertation KX2-391 (1 μM) and KX2 H means high concertation KX2-391 (2 μM). RLC, relative luciferase count. **(B)** Mlg cells were transiently transfected with selected siRNA designed against the Src gene for 48 h, and the mRNA of Src was detected by immunoblot and GAPDH was used as the endogenous reference protein. **(C)** After 24 h of siRNA transfection, the TOPFlash plasmid was transferred to the Mlg cells for 18 h, followed by Wnt3a/nintedanib treatment for 8 h and measurement with luciferase. RLC, relative luciferase count. **(D)** Schematic: Wnt-induced myofibroblast activation was attenuated by nintedanib by inhibiting Src activation and Y654-β-catenin phosphorylation. Each bar represents the mean ± SD of triplicate independent experiments.

To further verify whether the beneﬁcial effects provided by the administration of nintedanib were mediated through Src kinase, we used KX2-391, an effective Src inhibitor (Fallah-Tafti et al., 2011; Leng et al., 2016). Src inhibition resulted in a marked decrease in Wnt/β-catenin signaling, similar to that observed with nintedanib (**Figure 5A**). We then performed knockdown experiments by adding siRNA to Mlg cells. As shown in **Figure 5B**, siRNA, compared with a nonspecific control, effectively downregulated Src expression. Consistent with the results for nintedanib, Src knockdown induced a decrease in TOPFlash (**Figure 5C**). These results suggested the inhibition of Wnt/β-catenin signaling by nintedanib through the deactivation of Src kinase, but whether other potentially mechanisms that contribute to the inhibitory effect of nintedanib needed further prove.

## Discussion

Although IPF is an agnogenic disease, over the years, various hypotheses have been proposed for its pathogenesis. It has been documented that the Wnt/β-catenin signaling pathway is aberrantly activated, and the protein levels of Wnt isoforms increase in the lungs of IPF patients and BLM-treated mice (Chilosi et al., 2003; Konigshoff et al., 2009). One of the mechanisms affecting Wnt signaling is the phosphorylation of tyrosine at residue 654 in β-catenin by Src kinase (Brembeck et al., 2006). The principle behind this mechanisms is that the reduced afﬁnity of p-β-catenin for E-cad leads to an increase in the cytoplasmic pool, which allows the translocation of p-β-catenin into the nucleus and the promotion of its target gene transcription (Roura et al., 1999).

Nintedanib is a specific inhibitor of tyrosine kinases that mitigates numerous steps in the initiation and progression of IPF (Wollin et al., 2019). Although other tyrosine kinases inhibitors, such as imatinib (Aono et al., 2005) and sorafenib (Chen et al., 2013), have been demonstrated to ameliorate BLM-induced pulmonary ﬁbrosis *in vivo*, their clinical results for IPF treatment have been negative. These findings indicated to us that nintedanib probably has other potential targets and exerts anti-fibrosis effects through multiple synergistic pathways.

In this study, we investigated whether nintedanib has an inhibitory effect on Wnt3a-induced myofibroblast activation. Experimental data showed that nintedanib attenuated Wnt3a-induced ColIa1, Fn, and α-SMA expression *in vitro*, suggesting that nintedanib treatment rebalanced the accumulation of Wnt3a-induced ECM components, which is crucial for the amelioration of pulmonary fibrosis. We further explored the inhibitory role of nintedanib on the Wnt signaling pathway *in vitro* and found that it could inhibit Wnt/β-catenin signaling *via* a luciferase assay and real-time PCR. As expected, we observed similar results in the BLM-induced mouse model of fibrosis. It has been reported that the shuttle of β-catenin into the nucleus and its binding to the transcription factor TCFs/LEF1 is vital in the activation of Wnt signaling (Kim et al., 2011). Consistent with this theory, nintedanib inhibited the Wnt/β-catenin pathway by preventing β-catenin entry into the nucleus.

As mentioned earlier, we demonstrated how nintedanib suppressed the Wnt/β-catenin pathway by attenuating the phosphorylation of β-catenin Y654. Wnt3a stimulation enhanced Src kinase activity and increased Y654-β-catenin protein expression, which were abolished by nintedanib treatment (**Figure 5D**). Further study showed that abolished Src kinase activity led to a downregulation of the Wnt/β-catenin signal, which is consistent with nintedanib treatment.

In summary, our data demonstrated that the antagonistic effect of nintedanib on Wnt3a-induced myofibroblast activation is due to the inhibition of Src kinases. This study explored potential anti-fibrotic mechanisms of nintedanib and explained the underlying mechanisms of IPF. However, further experiments are needed to prove whether nintedanib inhibits the Wnt pathway through other pathways.

## Data Availability Statement

All datasets generated for this study are included in the article/**Supplementary Material**.

## Ethics Statement

The animal study was reviewed and approved by the Institutional Animal Care and Use Committee (IACUC) of Nankai University (Permit No. SYXK 2014-0003).

## Author Contributions

Conception and design: CY, HZ, and XiaohL. Collection and assembly of data: XiaowL, RD, and HY. Data analysis and interpretation: SG, QJ, KH, and RL. Manuscript writing: XiaopL and LZ. All authors read and approved the final manuscript.

## Funding

This study was supported by the National Science and Technology Major Projects for “Major New Drugs Innovation and Development” [Grant 2019ZX09201001], Fundamental Research Funds for the Central Universities, Nankai University [Grant 63191213, Grant 63191357], and Tianjin Science and Technology Project [Grant No. 17PTSYJC00070].

## Conflict of Interest

The authors declare that the research was conducted in the absence of any commercial or financial relationships that could be construed as a potential conflict of interest.

## Abbreviations

IPF, Idiopathic pulmonary fibrosis; PF, pulmonary fibrosis; ECM, extracellular matrix; Fn, fibronectin; ColI, collagen-I; α-SMA, α-smooth muscle actin; TCF, T-cell factor; LEF, lymphocyte enhance factor; E-Cad, E-cadherin; HGF, hepatocyte growth factor; TGF-β, transform growth factor-β; VEGFR, vascular endothelial growth factor receptor; FGFR, fibroblast growth factor receptor; PDGFR, platelet-derived growth factor receptor; FVC, forced vital capacity; GAPDH, glyceraldehyde-3-phosphate dehydrogenase; RIPA, radioimmunoprecipitation assay; BCA, bicinchoninic acid; PVDF, polyvinylidene fluoride; PBS, phosphate-buffered saline.
