# Supplementary material for: Nintedanib Inhibits Wnt3a-Induced Myofibroblast Activation by Suppressing the Src/β-Catenin Pathway
Source: Front Pharmacol. 2020 Mar 16;11:310. doi: 10.3389/fphar.2020.00310 (PMC7087487; doi:10.3389/fphar.2020.00310)

S1

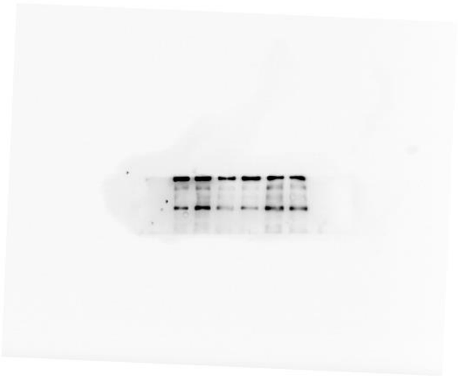

S2

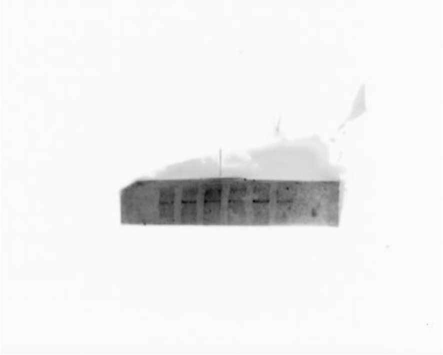

S3A

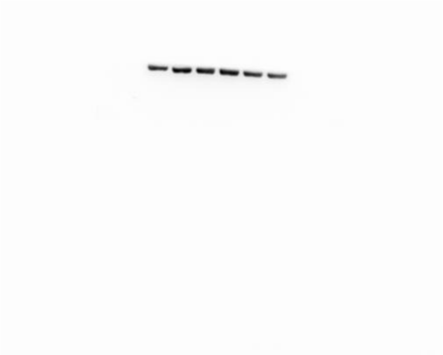

S3B

S4A

S4B

S5A

S5B

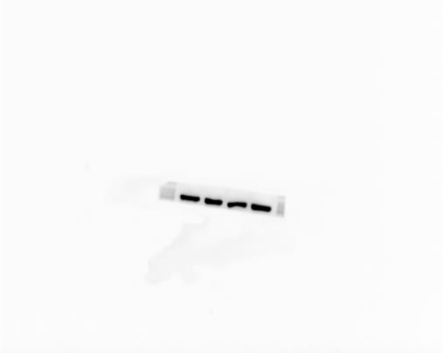

S6A

S6B

S6C

S7A

S7B

S7C

S8A

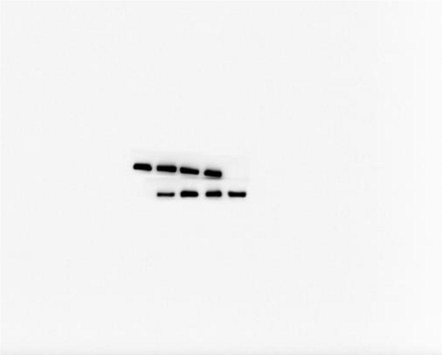

S8B

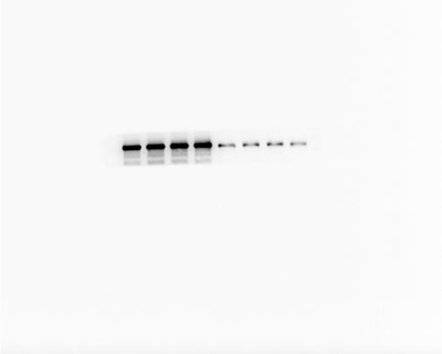

S8C

S9A

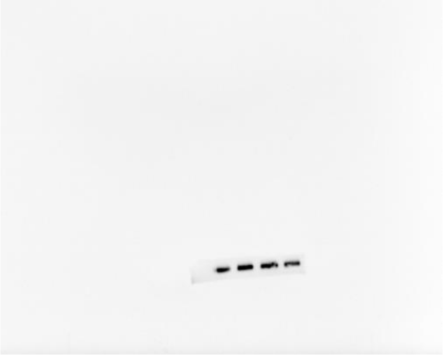

S9B

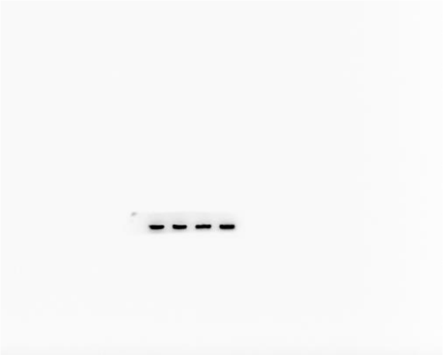

S9C

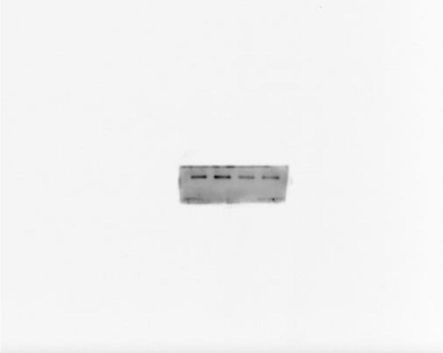

S9D

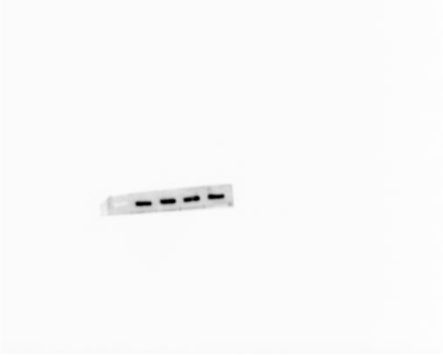

S9E

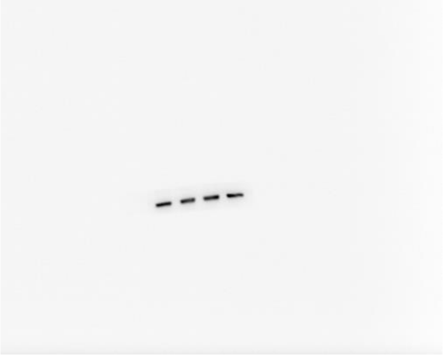

S10A

S10B

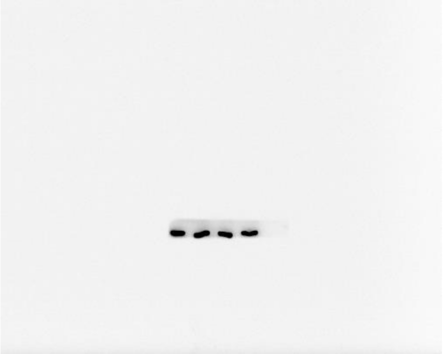

S10C

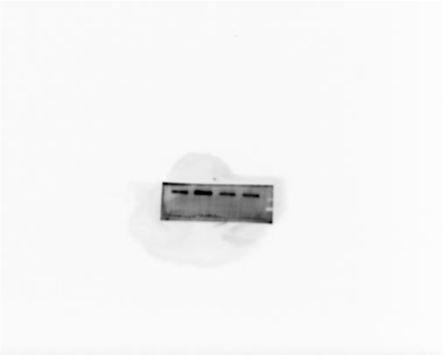

S10D

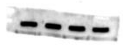

S10E

S11A

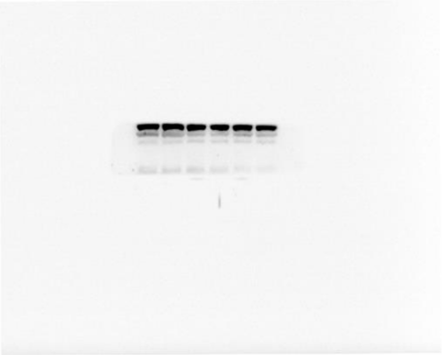

S11B

S11C

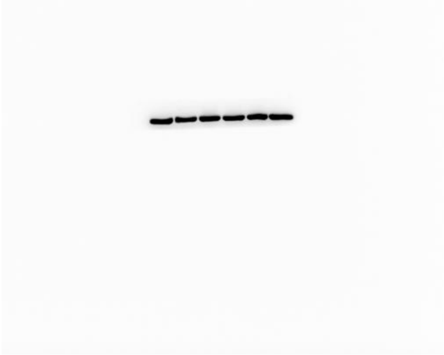

S12A

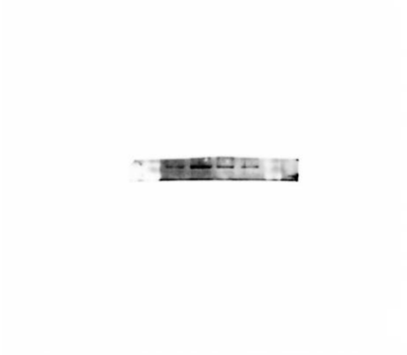

S12B

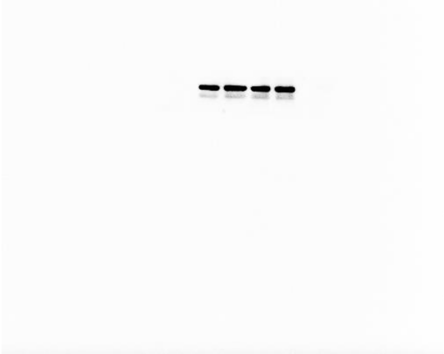

S12C

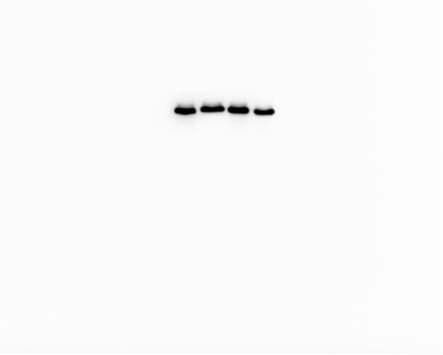

S13A

S13B

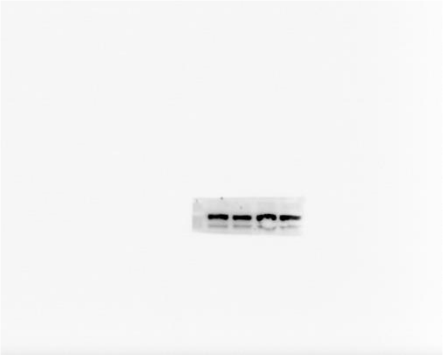

S13C

S13D

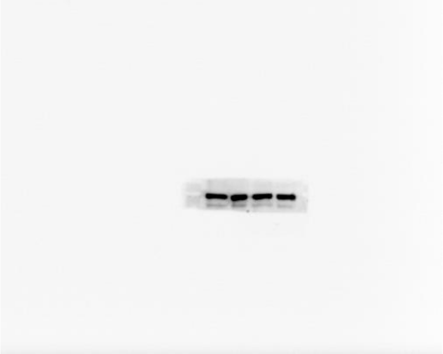

S13E

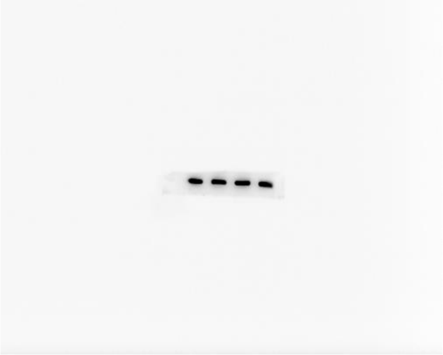

S14A

S14B

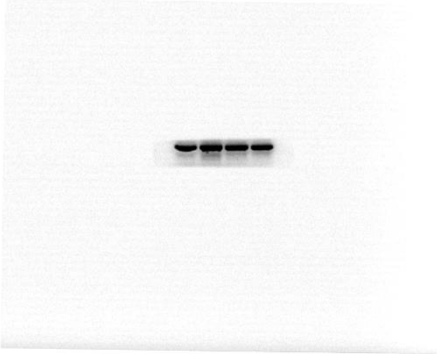

S14C

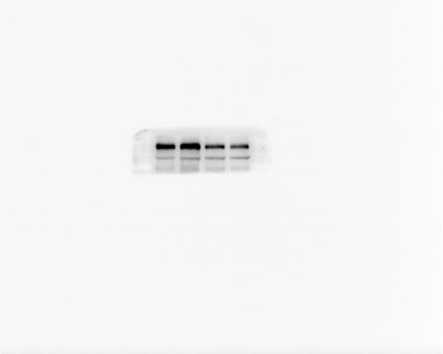

S14D

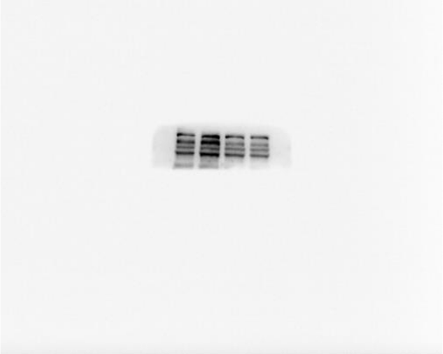

S14E

S15A

S15B

S15C

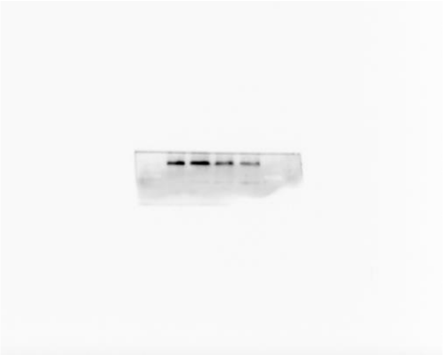

S15D

S15E

S16A

S16B

S16C

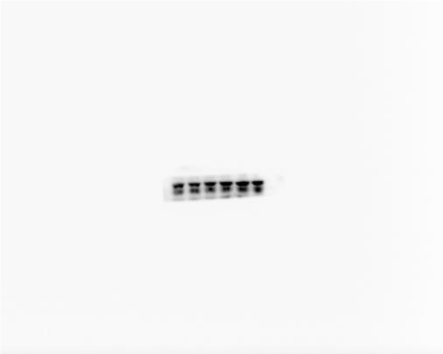

S16D

S16E

S17A

S17B

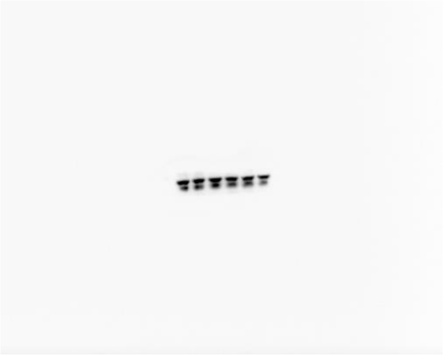

S17C

S17D

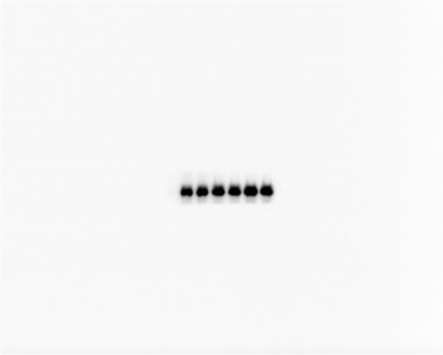

S17E

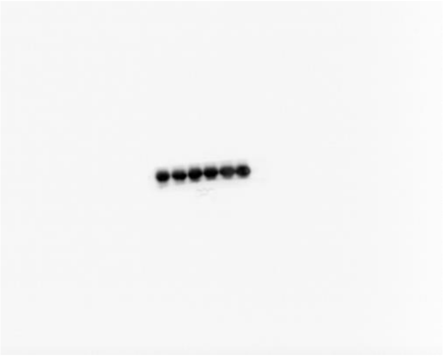

S18A

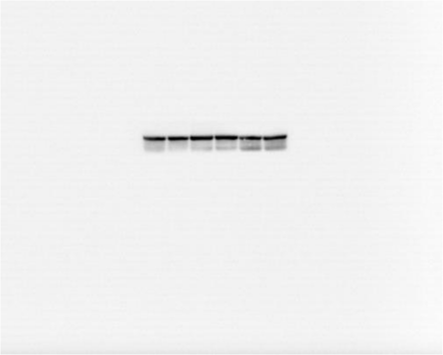

S18B

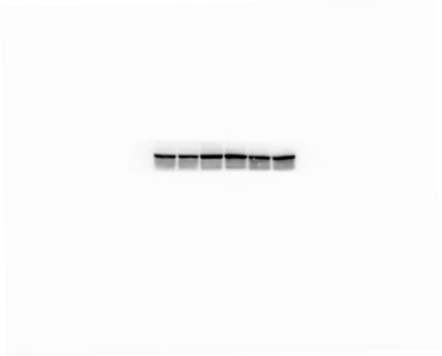

S18C

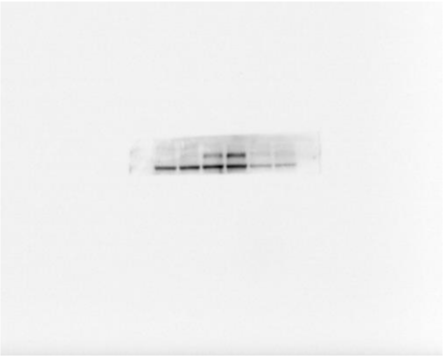

S18D

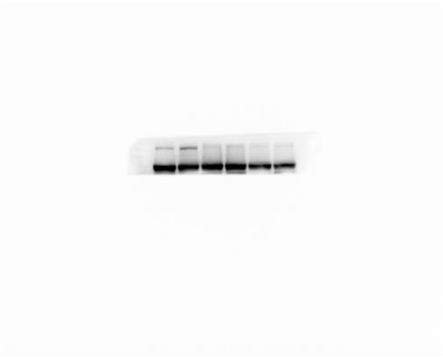

S18E

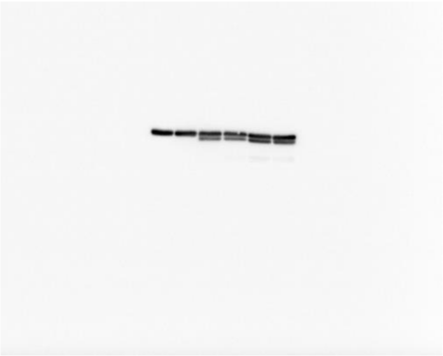

S19A

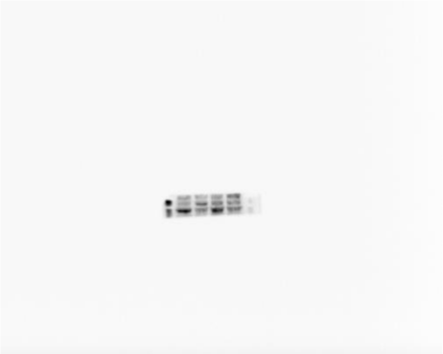

S19B

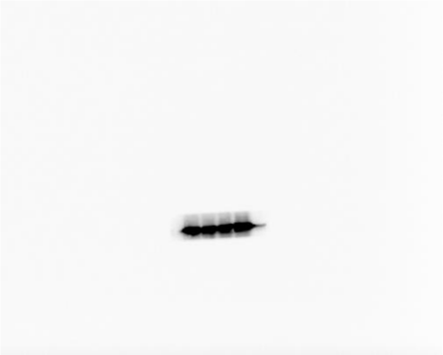

S20A

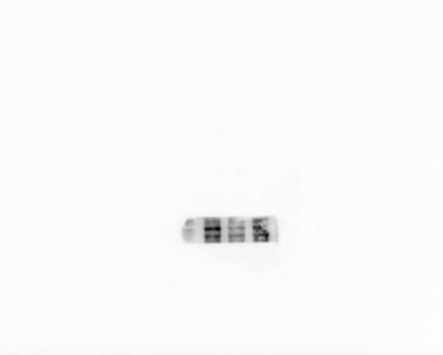

S20B

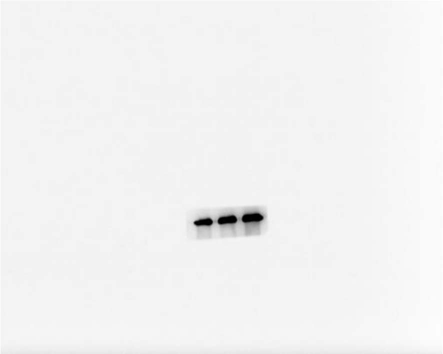

S21A

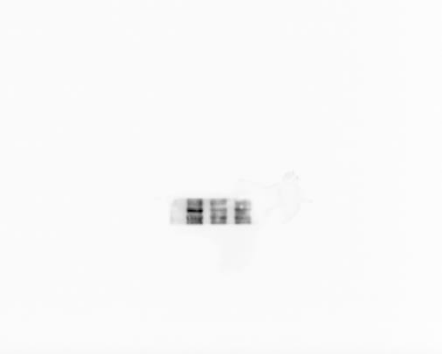

S21B

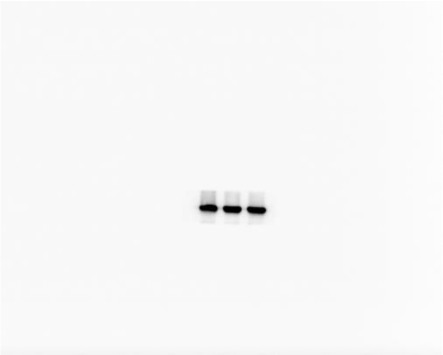

Supplement: Supplementary file 1 [file DataSheet_1.pdf]
