# Supplementary material for: Nintedanib Inhibits Wnt3a-Induced Myofibroblast Activation by Suppressing the Src/β-Catenin Pathway
Source: Front Pharmacol. 2020 Mar 16;11:310. doi: 10.3389/fphar.2020.00310 (PMC7087487; doi:10.3389/fphar.2020.00310)
Supplement: Supplementary file 2 [file DataSheet_2.pdf]

Supplementary Material

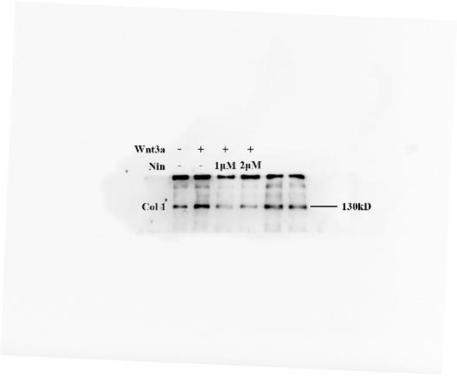

Supplementary Figure 1. The entire original gel of Col 1 in Figure 1E. The last two electrophoresis bands are the effects of other compound on the Col 1 protein level and are not relevant to this paper.

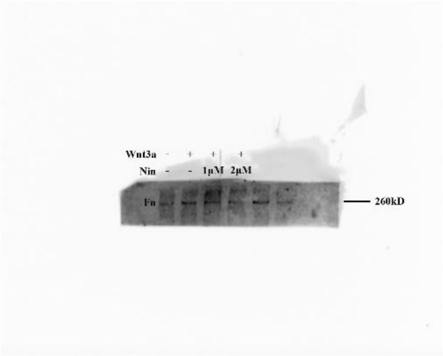

Supplementary Figure 2. The entire original gel of Fn in Figure 1E. The last two electrophoresis bands are the effects of other compound on the Fn protein level and are not relevant to this paper.

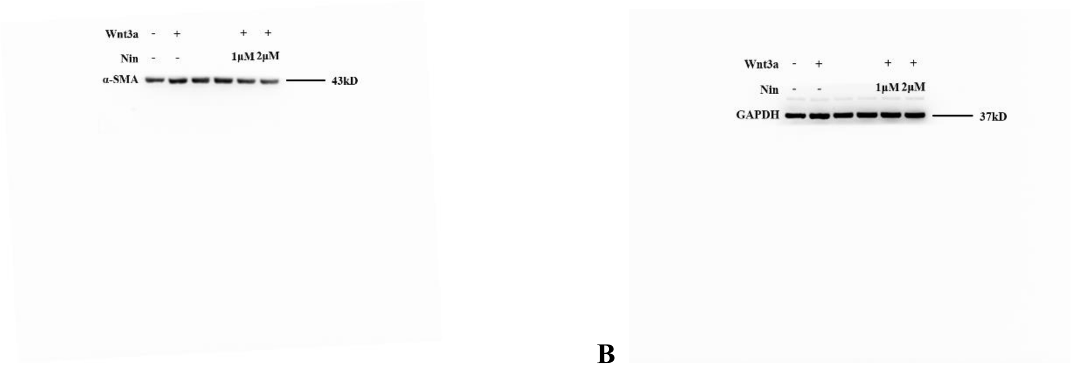

A **B**

Supplementary Figure 3. The entire original gel of (**A**) α-SMA and (**B**) GAPDH in Figure 1E. The middle two electrophoresis bands are the effects of other compound on the α-SMA protein level and are not relevant to this paper.

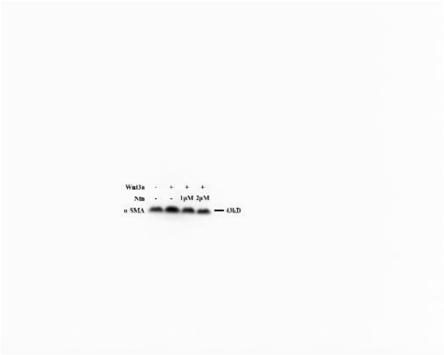

A

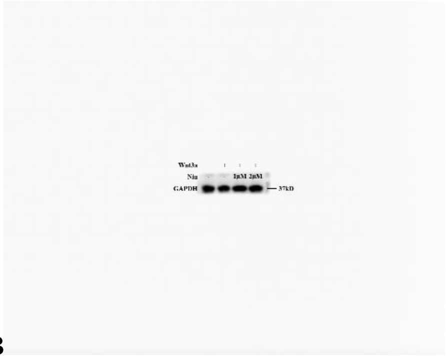

B

Supplementary Figure 4. The entire original gel of (**A**) α-SMA and (**B**) GAPDH which are not shown in Figure 1E.

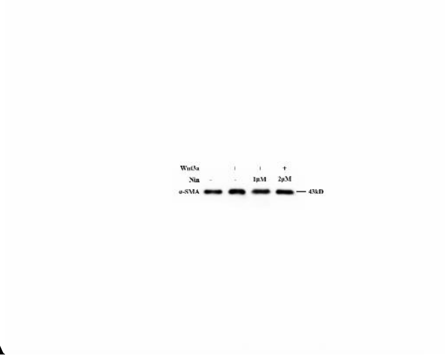

A

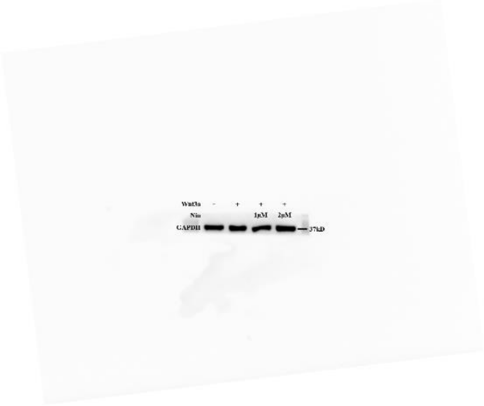

B

Supplementary Figure 5. The entire original gel of (**A**) α-SMA and (**B**) GAPDH which are not shown in Figure 1E. Figure 1F represent the calculate results of S3, S4 and S5.

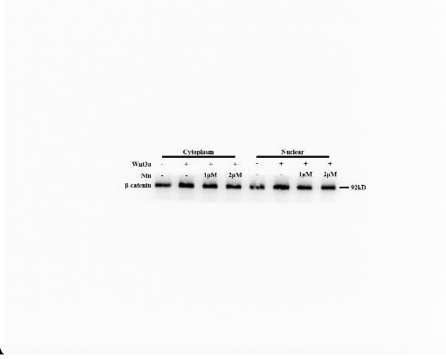

A

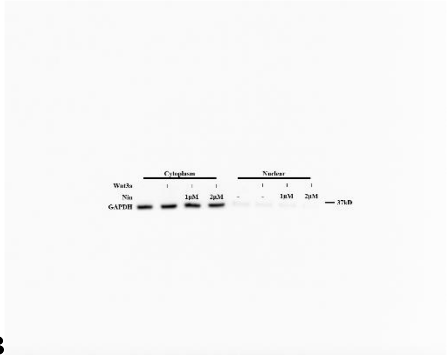

B

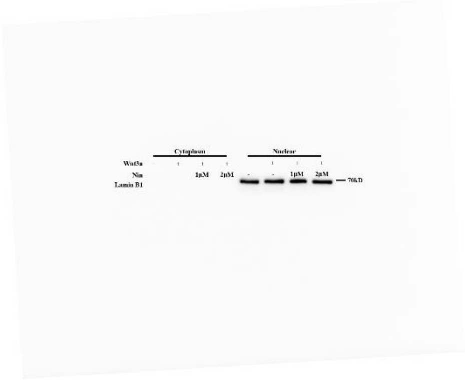

C

Supplementary Figure 6. The entire original gel of (**A**) β-catenin (**B**) GAPDH and (**C**) Lamin B in Figure 3A.

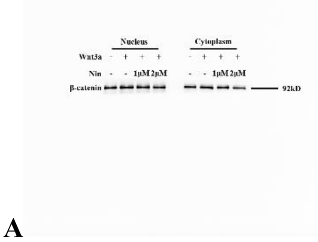

A

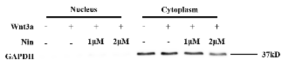

B

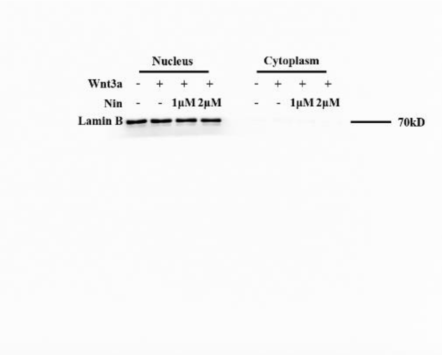

C

Supplementary Figure 7. The entire original gel of (**A**) β-catenin (**B**) GAPDH and (**C**) Lamin B which are not shown in Figure 3A. These three strips were obtained by twice western blot, but the samples and the loading amount were identical.

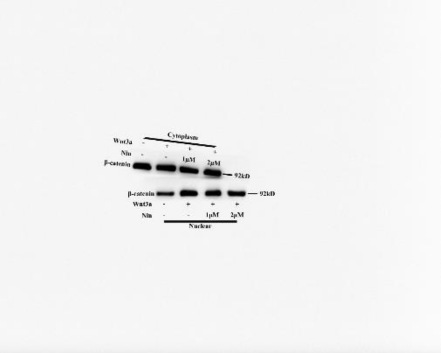

A

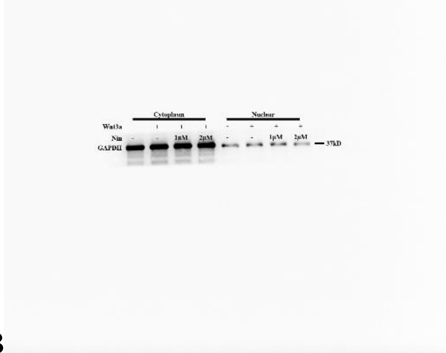

B

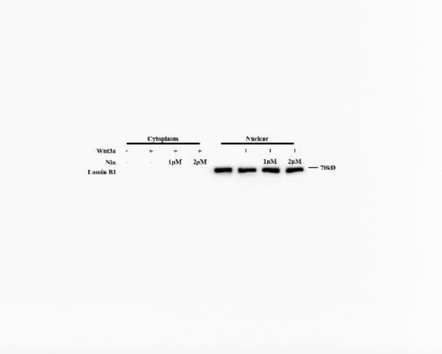

C

Supplementary Figure 8. The entire original gel of (**A**) β-catenin (**B**) GAPDH and

(**C**) Lamin B which are not shown in Figure 3A. Figure 3B-C represent the calculate results of S6, S7 and S8.

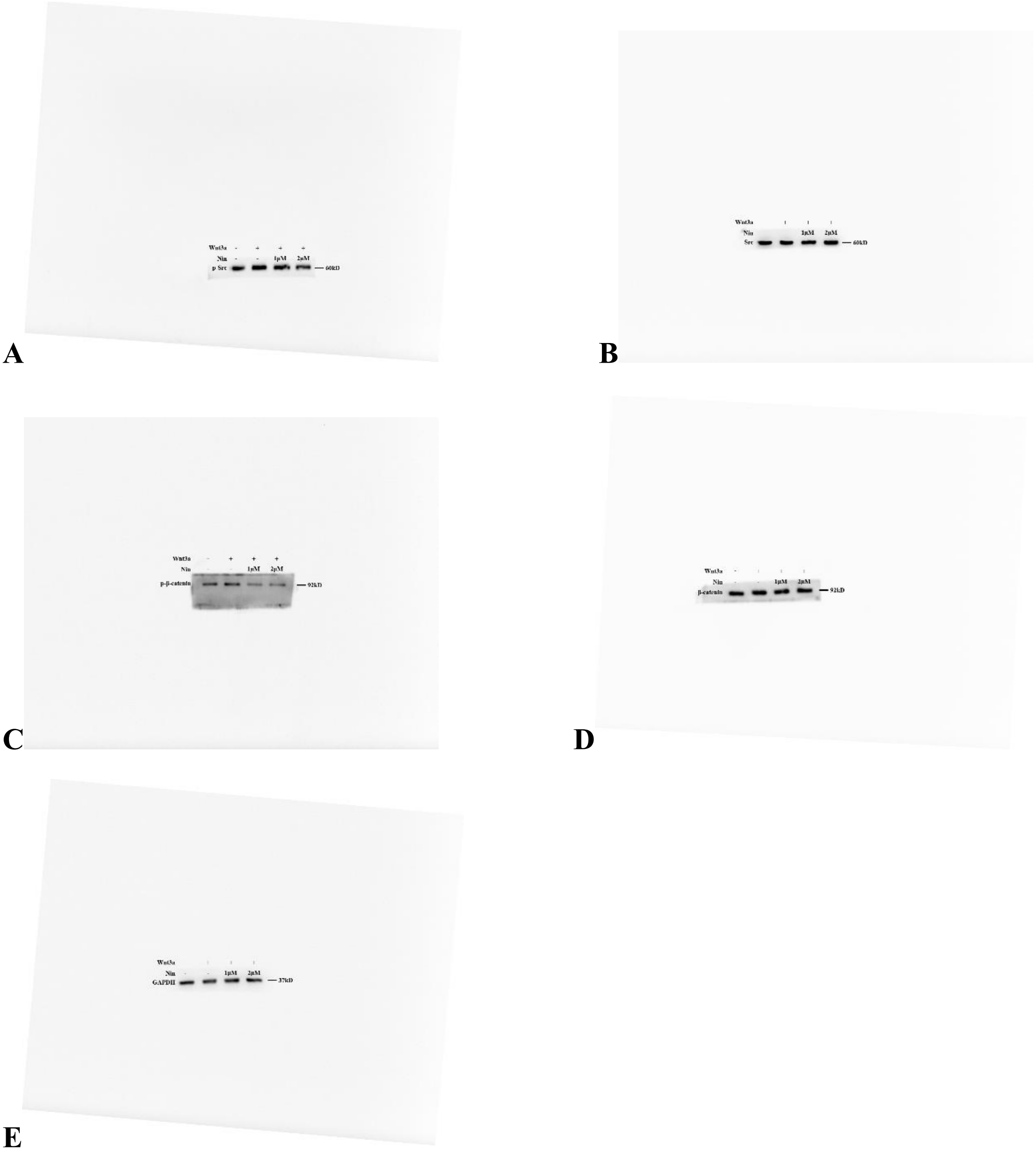

Supplementary Figure 9. The entire original gel of (**A**) p-Src, (**B**) Src, (**C**) p-β-catenin, (**D**) β-catenin and (**E**) GAPDH in Figure 4A.

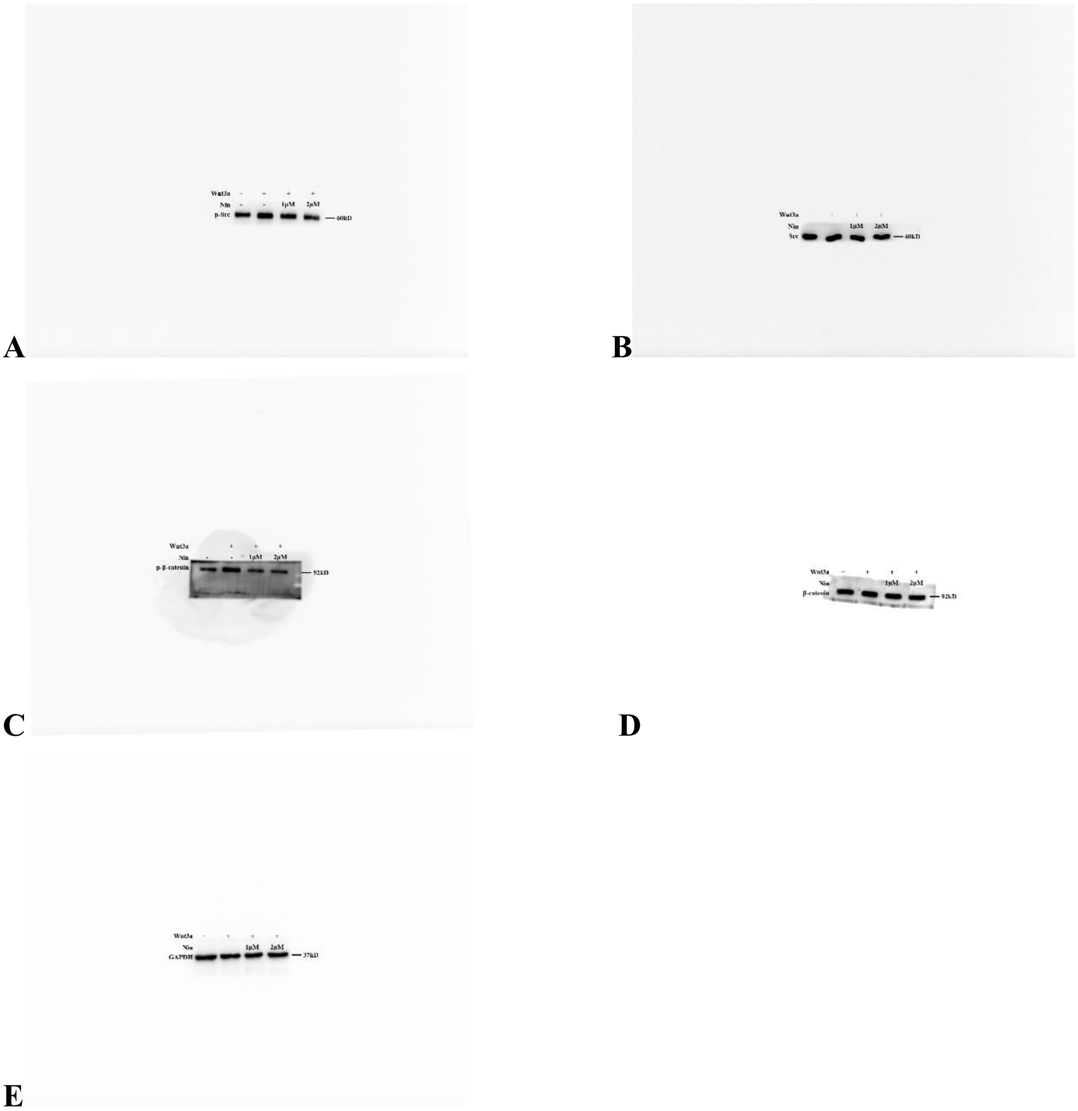

Supplementary Figure 10. The entire original gel of (**A**) p-Src, (**B**) Src, (**C**) p-β-catenin, (**D**) β-catenin and (**E**) GAPDH in Mlg cells which are not shown in Figure 4A.

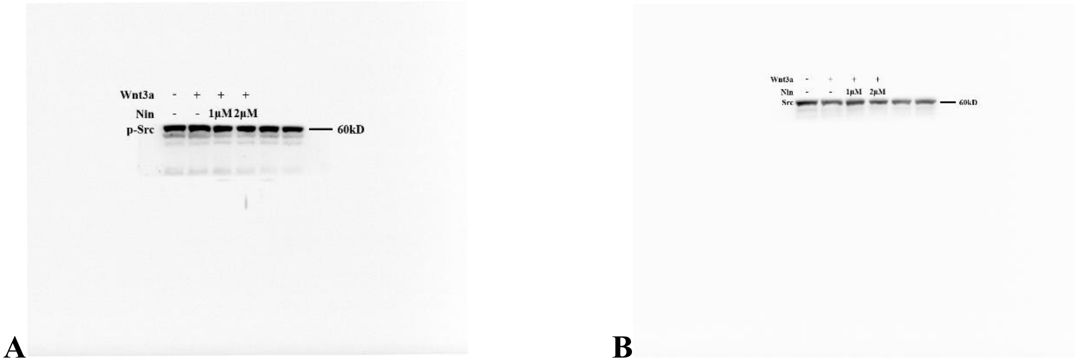

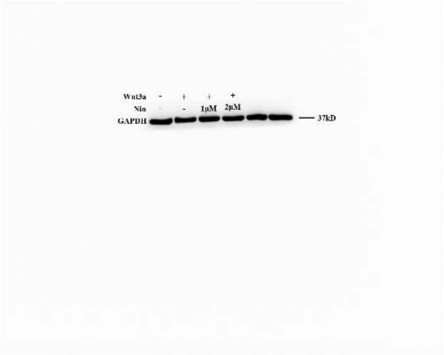

C

Supplementary Figure 11. The entire original gel of (**A**) p-Src, (**B**) Src and (**C**) GAPDH which are not shown in Figure 4A. The last two electrophoresis bands are the effects of other compound on the p-Src and Src protein levels and are not relevant to this paper.

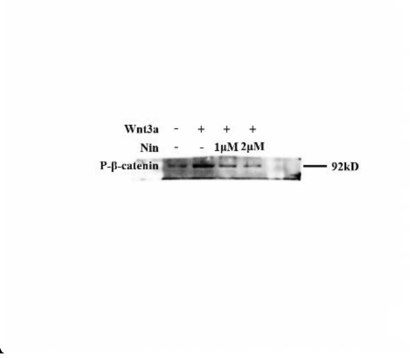

A

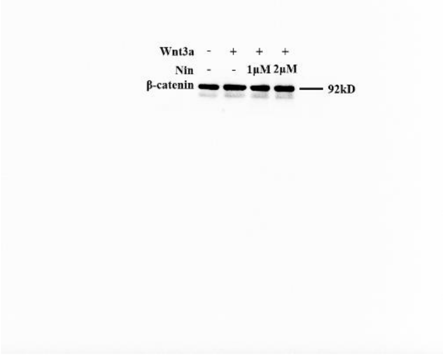

B

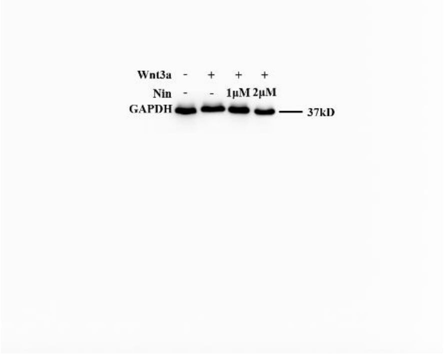

C

Supplementary Figure 12. The entire original gel of (**A**) p-β-catenin, (**B**) β-catenin and (**C**) GAPDH which are not shown in Figure 4A. For the consistency and alignment of data, GAPDH is not involved in Figure 4A. The scatterplots in Figure 4A represent the calculate results of S9-12.

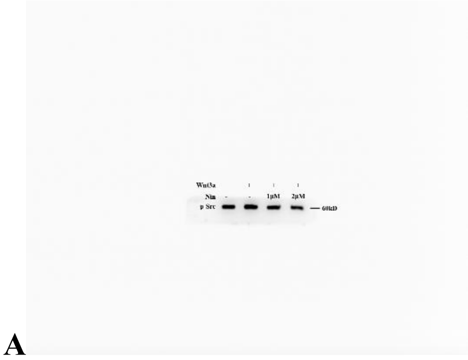

A

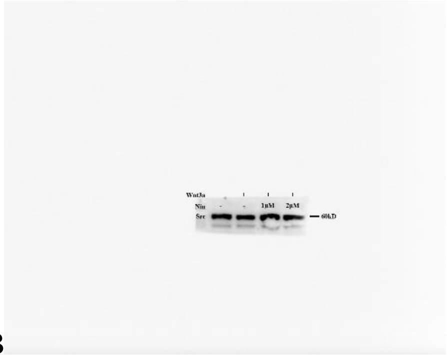

B

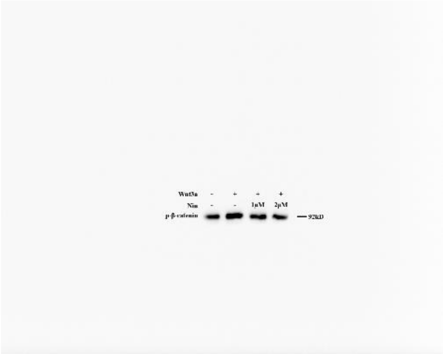

C

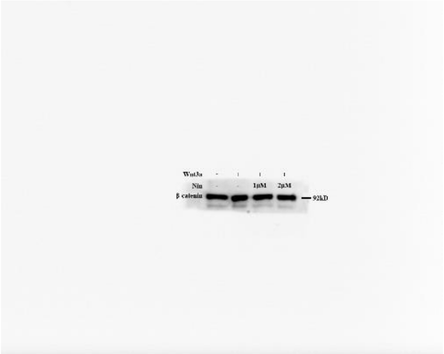

D

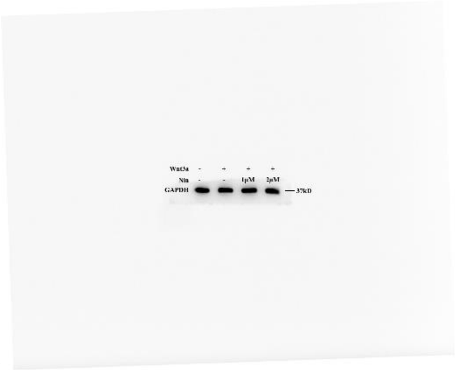

E

Supplementary Figure 13. The entire original gel of (**A**) p-Src, (**B**) Src, (**C**) p-β-catenin, (**D**) β-catenin and (**E**) GAPDH in Figure 4B.

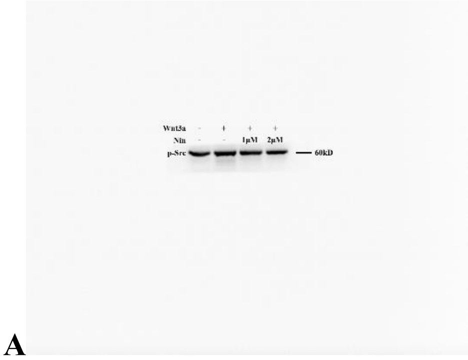

A

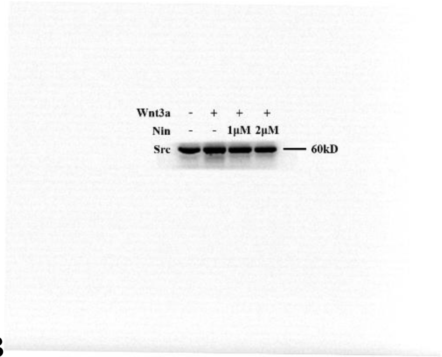

B

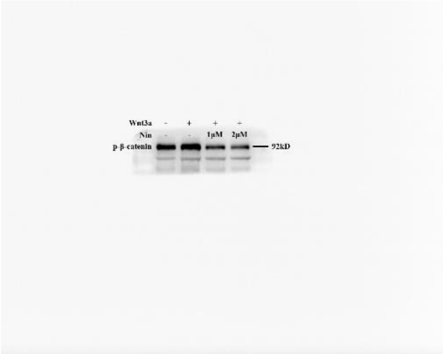

C

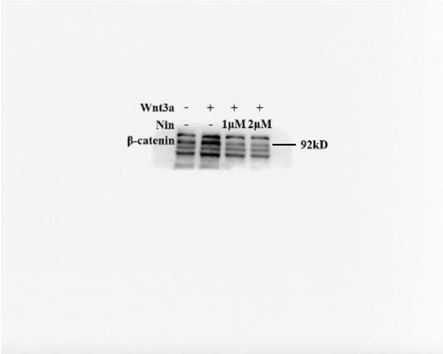

D

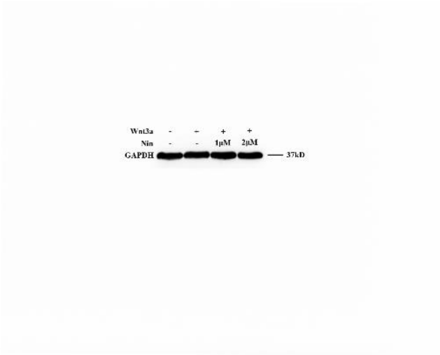

E

Supplementary Figure 14. The entire original gel of (**A**) p-Src, (**B**) Src, (**C**) p-β-catenin, (**D**) β-catenin and (**E**) GAPDH in PPF cells which are not shown in Figure 4B.

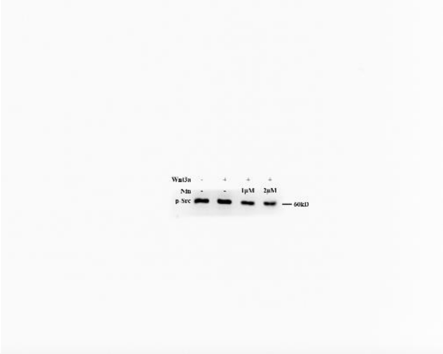

A

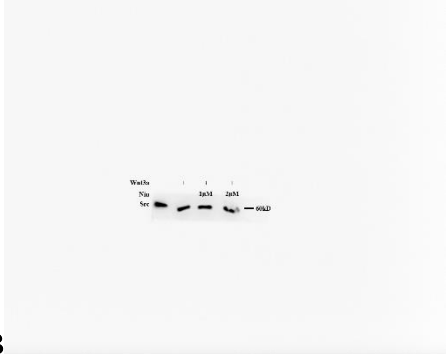

B

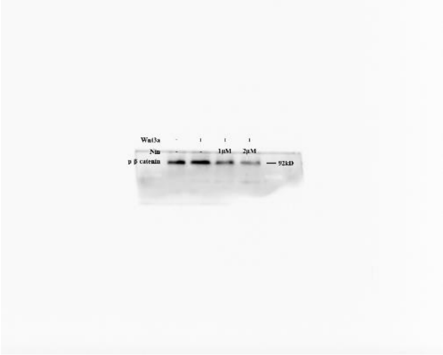

C

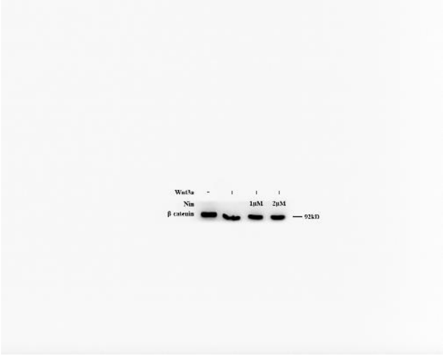

D

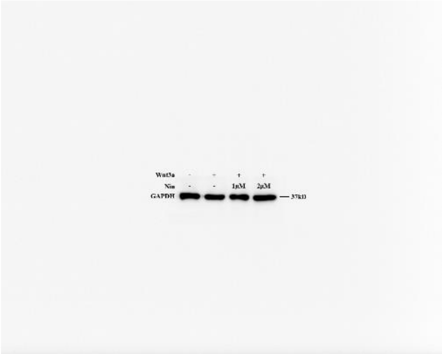

E

Supplementary Figure 15. The entire original gel of (**A**) p-Src, (**B**) Src, (**C**) p-β-catenin, (**D**) β-catenin and (**E**) GAPDH in PPF cells which are not shown in Figure 4B. The scatterplots in Figure 4B represent the calculate results of S13-15.

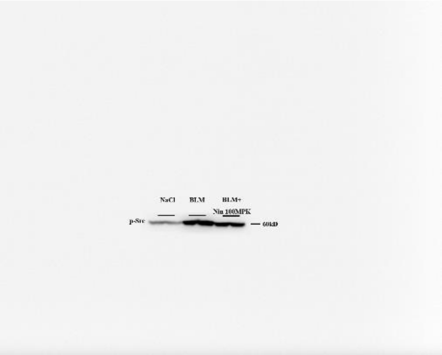

A

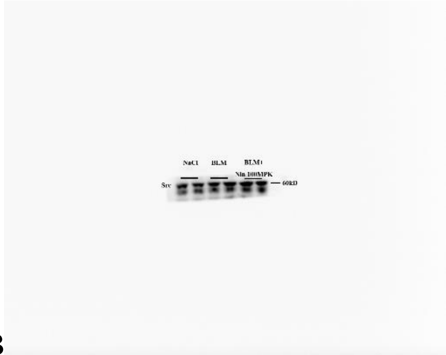

B

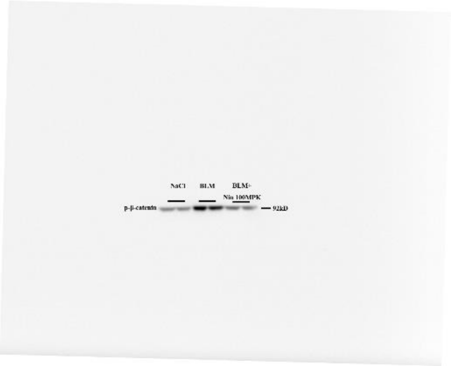

C

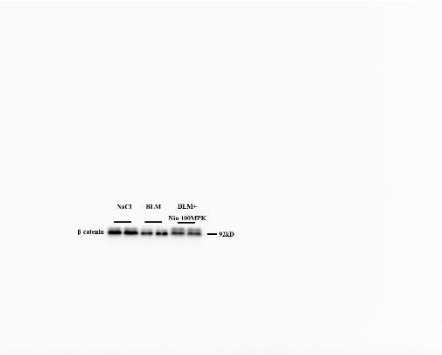

D

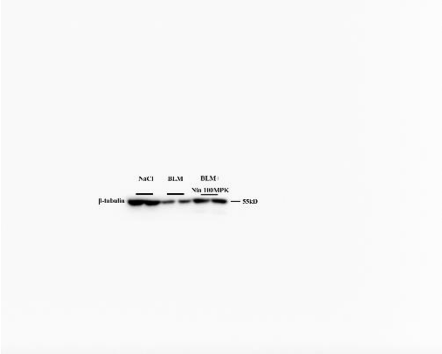

E

Supplementary Figure 16. The entire original gel of (**A**) p-Src, (**B**) Src, (**C**) p-β-catenin, (**D**) β-catenin and (**E**) GAPDH in Figure 4C.

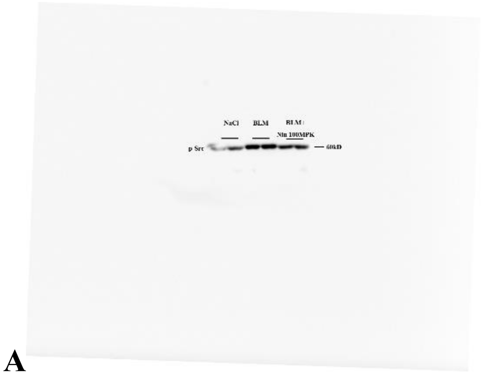

A

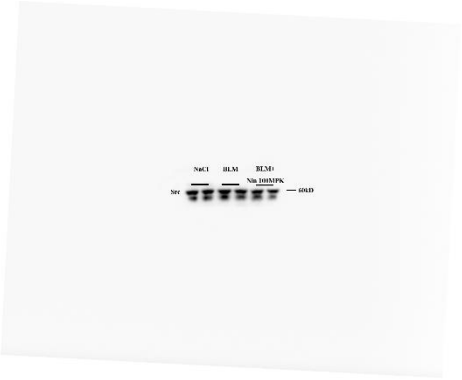

B

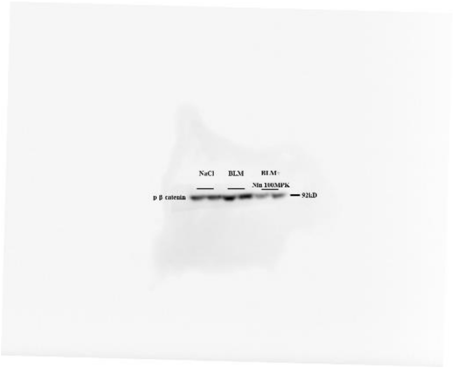

C

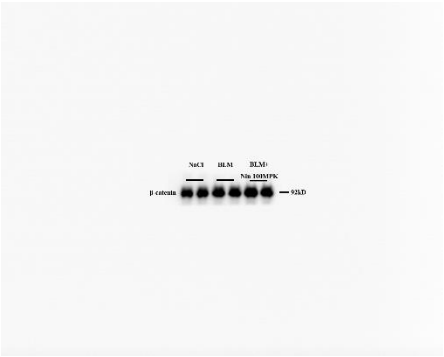

D

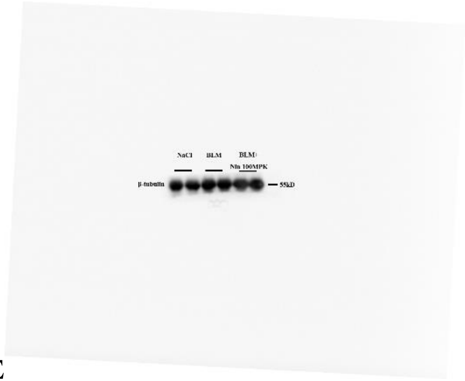

E

Supplementary Figure 17. The entire original gel of (**A**) p-Src, (**B**) Src, (**C**) p-β-catenin, (**D**) β-catenin and (**E**) GAPDH in lung tissues which are not shown in Figure 4C.

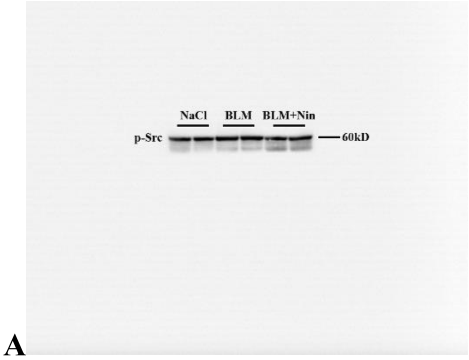

A

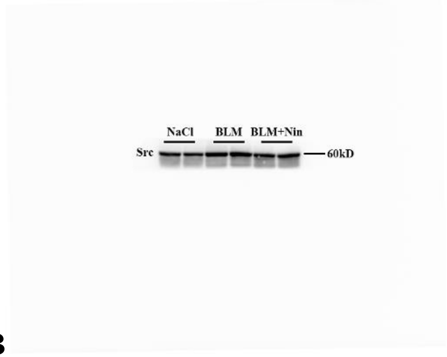

B

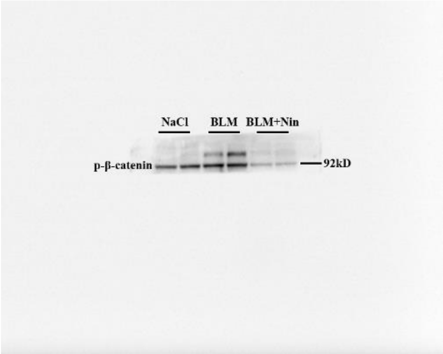

C

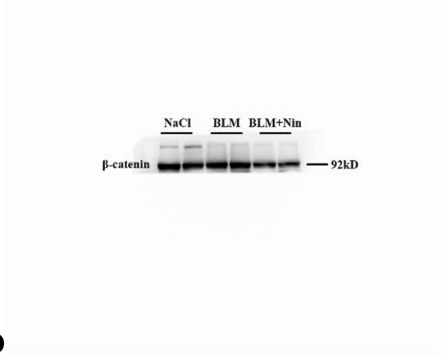

D

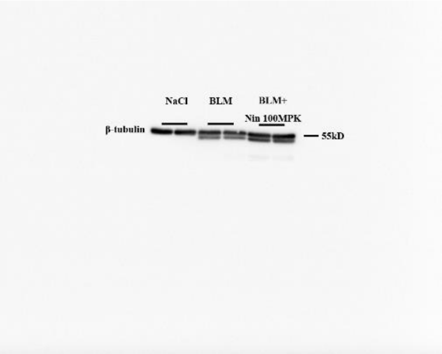

E

Supplementary Figure 18. The entire original gel of (**A**) p-Src, (**B**) Src, (**C**) p-β-catenin, (**D**) β-catenin and (**E**) GAPDH in lung tissues which are not shown in Figure 4C. The scatterplots in Figure 4C represent the calculate results of S16-18.

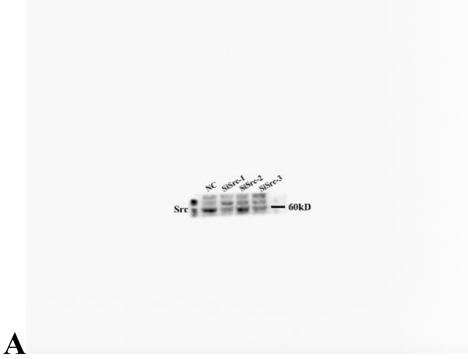

A

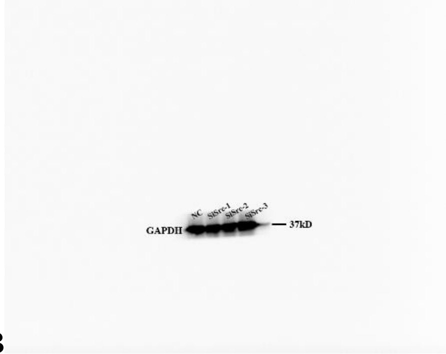

B

Supplementary Figure 19. The entire original gel of (**A**) Src, (**B**) GAPDH which are not shown in Figure 5B. We synched 3 SiRNA targeted Src kinase and found that picked SiSrc-1 and SiSrc-3 for further comparison.

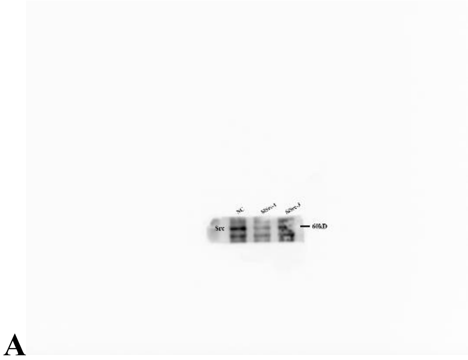

A

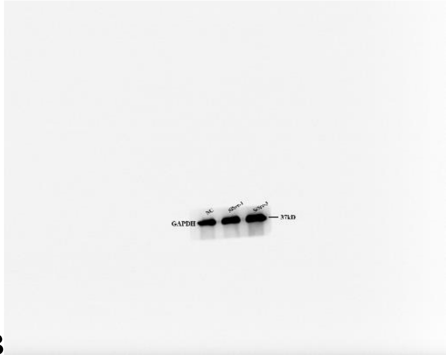

B

Supplementary Figure 20. The entire original gel of (**A**) Src, (**B**) GAPDH which are shown in Figure 5B.

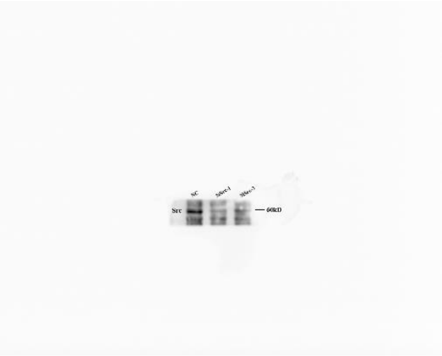

A

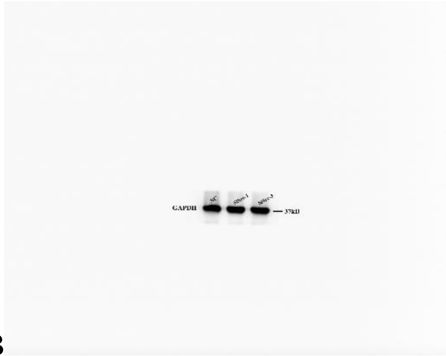

B

Supplementary Figure 21. The entire original gel of (**A**) Src, (**B**) GAPDH which are not shown in Figure 5B. We finally selected SiSrc-1 for subsequent experiments. The scatterplot in Figure 5B represents the calculate results of S19-21.
