# Supplementary figures and images for: Nintedanib Inhibits Wnt3a-Induced Myofibroblast Activation by Suppressing the Src/β-Catenin Pathway
Source: Front Pharmacol. 2020 Mar 16;11:310. doi: 10.3389/fphar.2020.00310 (PMC7087487; doi:10.3389/fphar.2020.00310)

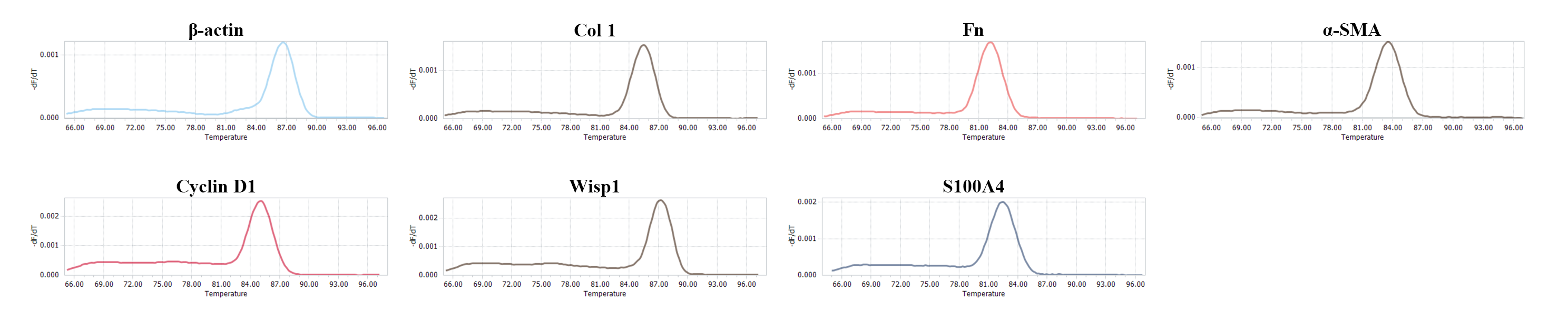

Supplement: Supplementary file 4 [file Image_1.tif]
